# Understanding pain in women with polyendocrine metabolic ovarian syndrome: health risks and treatment effectiveness

**DOI:** 10.7554/eLife.103875

**Published:** 2026-08-28

**Authors:** Tess Cherlin, Stephanie Mohammed, Samantha Strydesky, Sasha Ottey, Katherine Sherif, Shefali Setia Verma

**Affiliations:** 1 Department of Pathology and Laboratory Medicine, Perelman School of Medicine Philadelphia United States; 2 PCOS Challenge: The National Polycystic Ovary Syndrome Association Atlanta United States; 3 Department of Medicine, Sidney Kimmel Medicine College, Thomas Jefferson University Philadelphia United States; Icahn School of Medicine at Mount Sinai United States; McGill University Canada

**Keywords:** PCOS, PMOS, pain, long term outcomes, EHR, prevelance, Human

## Abstract

**Background:**

Polycystic ovary syndrome (PCOS), recently renamed as polyendocrine metabolic ovarian syndrome (PMOS), is a prevalent endocrine disorder in women, often accompanied by various symptoms, including significant pain, such as dysmenorrhea, abdominal, and pelvic pain, which remains underexplored.

**Methods:**

This retrospective study examines electronic health records (EHR) data to assess the prevalence of pain in women with PMOS. Conducted in January 2026, using data from 120 Health Care Organizations within the TriNetX Global Network, the study involved 103,675,738 women from diverse racial backgrounds. The analysis focused on the prevalence of pain among women with PMOS, both overall and in those prescribed PMOS-related medications. Relative risk ratios (RR) were calculated for future health outcomes and stratified by self-reported race.

**Results:**

The study found that 20.67% of women with PMOS experienced pain, with the highest prevalence among Black or African American (32.70%) and White (30.78%) populations. Both the PMOS and PMOS and Pain cohorts exhibited increased RR for various health conditions, with significant differences noted across racial groups for infertility, ovarian cysts, obesity, and respiratory diseases. Additionally, women with PMOS who were treated with PMOS-related medications showed a decrease in pain diagnoses following treatment.

**Conclusions:**

This study highlights the critical need to address pain in the diagnosis and management of PMOS due to its significant impact on patient health outcomes.

**Funding:**

Tess Cherlin was supported by NIH | National Institute of General Medical Sciences (NIGMS) (grant # K12GM081259 (HHS)).

## eLife digest

PMOS (polyendocrine metabolic ovarian syndrome), previously known as PCOS (polycystic ovary syndrome), is a condition affecting the body's metabolism, reproductive system, hormones and skin. It affects an estimated 8–13% of women. Healthcare professionals diagnose PMOS based on a combination of irregular menstrual periods, signs of higher levels of hormones called androgens, and changes in the ovaries, after ruling out other causes. The condition is associated with a wide range of symptoms that can have a major impact on quality of life.

Pain has been linked to PMOS, but little is known about the different types of pain women experience, such as menstrual pain versus abdominal or pelvic pain. Even less is known about the long-term health effects of pain in women with PMOS, or whether these effects differ across racial groups. To address these questions, Cherlin et al. analysed electronic health records, which contain information on patients' diagnoses, medications and procedure codes. This allowed them to examine long-term health patterns in a very large population.

The researchers investigated whether women with PMOS experience more pain than women without the condition. They also examined whether women with both PMOS and pain were at greater risk of developing future health problems than women with PMOS overall. In addition, they explored differences in pain across self-reported racial groups and assessed whether commonly prescribed PMOS medications influenced pain symptoms.

Using electronic health records data from more than 100 million women worldwide, Cherlin et al. found that 21% of women with PMOS experienced pain, with the highest prevalence among Black or African American women (33%). Overall, women with PMOS were at increased risk of developing a range of health conditions, including infertility, type 2 diabetes and liver disease. Women with both PMOS and pain had an even greater risk of several conditions, including ovarian cysts, anxiety, depression, acute pharyngitis and gastroesophageal reflux disease. Women who were prescribed common PMOS medications experienced reduced pain after treatment.

These findings could inform improved care for women with PMOS, particularly the one in five who experience pain. They highlight the need for routine pain assessment and management to become part of standard PMOS care. The results also suggest that racial differences should be considered when developing more personalised treatment strategies, as the impact of PMOS and pain varied across self-reported racial groups. Overall, this research provides new insight into the relationship between PMOS and pain and may help improve diagnosis, treatment and long-term patient care.

## Introduction

According to the WHO, polycystic ovary syndrome (PCOS) recently renamed to polyendocrine metabolic ovarian (Teede et al., 2026) syndrome (PMOS) affects approximately 8–13% of women of reproductive age, with an alarming 70% of affected individuals remaining undiagnosed globally (World Health Organization, 2023, June 28, 2023). The assessment of PMOS has been substantiated by multiple guidelines (Azziz et al., 2016; Teede et al., 2010) and has undergone refinement since its initial description by Stein and Leventhal, 1935. Standard diagnostic criteria have evolved through international efforts, including conferences convened by the National Institutes of Health (NIH) in 1990 (Zawadri, 1992), the ESHRE/ASRM-sponsored PMOS consensus workshop group in Rotterdam in 2003 and 2004 (ESHRE/ASRM-Sponsored PCOS Consensus Workshop Group, 2004), and the International Evidence-based Guideline for the Assessment and Management of Polycystic Ovary Syndrome in 2018, most recently updated in 2023 (Mousa and Tay, 2023; Teede et al., 2018).

Recommendations for assessing PMOS encompass a multifaceted approach, including the evaluation of irregular menstrual cycles, ovulatory dysfunction, biochemical and clinical hyperandrogenism, ultrasound findings, serum Anti-Müllerian hormone (AMH) levels, and various other factors such as ethnic disparities, cardiovascular disease risk, menopausal status, impaired glucose tolerance, and risk of type 2 diabetes mellitus (T2DM; Mousa and Tay, 2023; Teede et al., 2018). Additionally, screening and managing psychological manifestations, implementing lifestyle interventions, and adhering to pharmacological treatment principles are integral aspects of PMOS management (Mousa and Tay, 2023; Teede et al., 2018). While the diagnostic criteria for PMOS primarily focus on reproductive and metabolic manifestations, the substantial burden of pain experienced among women with PMOS is a critical factor that warrants effective prevention and management of the disease.

The assessment of pain in PMOS necessitates a multidimensional approach, incorporating self-reported scales, clinical evaluation, and possibly imaging techniques to elucidate the underlying etiology and severity. Several commonly utilized assessment tools incorporate evaluations of pain among women with PMOS. The Polycystic Ovary Syndrome Health-Related Quality of Life Questionnaire (PCOSQ), developed by Cronin et al., 1998, assesses various domains, including painful menstrual cycles. Additionally, the SF-36 scale examines eight dimensions of health, including bodily pain (McHorney et al., 1993). Women with PMOS across diverse demographic backgrounds have consistently reported lower SF-36 scores, specifically in the domain of bodily pain (Drosdzol et al., 2007; Elsenbruch et al., 2003; Hahn et al., 2005; Li et al., 2011). Furthermore, the Menorrhagia Outcomes Questionnaire, developed by Lamping et al., 1998, evaluates both heavy menstrual bleeding (HMB) and the associated pain. Despite the validation of these instruments, they may not comprehensively capture key symptoms expressed by patients with PMOS, especially those related to dysmenorrhea, abdominal, or pelvic pain. Insufficient data exist to highlight the prevalence of pain reported by women both before and after a PMOS diagnosis, as well as any associations between this pain and the condition itself and its long-term effects. To address this gap in research, we propose an investigation utilizing health records to shed light on this underexplored aspect of PMOS.

Electronic health records (EHRs) have become indispensable for managing vast amounts of clinical data, including patient demographics, medical history, medications, allergies, laboratory test results, vital signs, and imaging reports, as well as genetic information obtained from patient genomes when available. Given that EHRs contain comprehensive information about patient care, including the progression of signs and symptoms, severity, comorbidities, and treatments, they provide invaluable resources for conducting large-scale retrospective studies. EHR-based studies have been particularly valuable in assessing the prevalence of conditions that are often underdiagnosed or misdiagnosed in women (Kruse et al., 2018; Maletzky et al., 2022; Penrod et al., 2023). The temporal aspect of clinical events, such as the onset of symptoms, treatment administration, and follow-up visits, can also be mined from EHRs, providing crucial insights into disease trajectories and treatment efficacy (Zhao et al., 2017).

Pain, particularly in the context of PMOS, remains an underexplored area of research. By leveraging EHR data, we can identify women with PMOS who have reported dysmenorrhea, abdominal, and pelvic pain. The objective of this research is to use EHR and look at longitudinal data retrospectively to determine the pain reported by women with PMOS and to compare this to women without PMOS. The primary hypothesis of this study is that women with PMOS experience a higher prevalence of pain (including dysmenorrhea, abdominal pain, and pelvic pain) compared to women without PMOS, and this prevalence varies by racial groups. The hypothesis aims to investigate the prevalence of pain in women with and without pain. Our approach will provide insights into the relationship between pain symptoms and PMOS, contributing to a better understanding of the condition and potentially improving patient care.

## Methods

### Study design

The data used in this study was collected on January 9, 2026, from the TriNetX Global Network, which provided access to electronic medical records (diagnoses, procedures, medications, laboratory values, genomic information) from approximately 196,361,552 million patients from 172 healthcare organizations in 19 different countries. TriNetX has a rigorous quality control pipeline which can be found in the platform's documentation. Briefly, EHR data is received from Health Care Organizations (HCOs) in CSV format. TriNetX maps the data to a standard and controlled set of clinical terminology. Demographics data are mapped to HL7 administrative standards, diagnoses are represented by ICD codes, procedures are represented by ICD and CPT codes, and medications are mapped to RxNorm ingredients. The data is then transformed into a proprietary data format. Data cleaning is performed, and records that don’t meet the TriNetX quality standards are excluded. Quality checks are done for formatting, and records with missing required data are excluded. TriNetX does not impute or estimate clinical values to fill gaps in patients’ records, and there is no guarantee of data completeness.

### Cohort definitions

A retrospective cohort analysis was conducted for patients with PMOS and patients with PMOS and Pain. Patients who were identified as cases (met inclusion and exclusion criteria) were compared to their respective controls. Description of inclusion and exclusion criteria for each cohort can be found in Table 1.

**Table 1.** Inclusion and exclusion criteria for PMOS (top) and PMOS and Pain (bottom) cases and control cohorts.

	PMOS	No PMOS
Inclusion	Female	Female
	PCOS diagnosis (ICD 10 Code E28.2)	
	Irregular menstruation (ICD 10 Code N92.6) and hirsutism (ICD 10 Code L68.0) or irregular menstruation (ICD 10 Code N92.6) and androgen excess (ICD 10 Code)	
Exclusion	Endometriosis (ICD 10 Code N80)	PCOS diagnosis (ICD 10 Code E28.2)
	Uterine fibroids (ICD 10 Code D25, O34.1, O34.11, O34.10)	Irregular menstruation (ICD 10 Code N92.6) and hirsutism (ICD 10 Code L68.0)
	Polyp of corpus uteri (ICD 10 Code N84.0)	Irregular menstruation (ICD 10 Code N92.6) and androgen excess
	Pelvic inflammation (ICD 10 Code N73.9)	Endometriosis (ICD 10 Code N80), uterine fibroids (ICD 10 Code D25, O34.1), polyp of corpus uteri (ICD 10 Code N84.0), pelvic inflammation (ICD 10 Code N73.9), hypothyroidism (ICD 10 Code E03.8, E03.9), hyperprolactinemia (ICD 10 Code E22.1), adrenal hyperplasia (ICD 10 Code E27.8, Q89.1)
	**PMOS and Pain**	**PMOS**
Inclusion	Female	Female
	PCOS diagnosis (ICD 10 Code E28.2)	PCOS Diagnosis (ICD 10 Code E28.2)
	Irregular menstruation (ICD 10 Code N92.6) and hirsutism (ICD 10 Code L68.0) or irregular menstruation (ICD 10 Code N92.6) and androgen excess (ICD 10 Code)	Irregular menstruation (ICD 10 Code N92.6) and hirsutism (ICD 10 Code L68.0) or irregular menstruation (ICD 10 Code N92.6) and androgen excess (ICD 10 Code)
	Abdominal and pelvic pain (ICD Code 10 R10) or dysmenorrhea (ICD Code 10 N94.6)	
Exclusion	Endometriosis (ICD 10 Code N80), uterine fibroids (ICD 10 Code D25, O34.1, O34.11, O34.10), hypothyroidism (ICD 10 Code E03.8, E03.9), pelvic inflammation (ICD 10 Code N73.9), hyperprolactinemia (ICD 10 Code E22.1), adrenal hyperplasia (ICD 10 Code E27.8, Q89.1), polyp of corpus uteri (ICD 10 Code N84.0)	Endometriosis (ICD 10 Code N80), uterine fibroids (ICD 10 Code D25, O34.1, O34.11, O34.10), hypothyroidism (ICD 10 Code E03.8, E03.9), pelvic inflammation (ICD 10 Code N73.9), hyperprolactinemia (ICD 10 Code E22.1), adrenal hyperplasia (ICD 10 Code E27.8, Q89.1), polyp of corpus uteri (ICD 10 Code N84.0)
		Abdominal and pelvic pain (ICD Code 10 R10) or dysmenorrhea (ICD Code 10 N94.6)

For the PMOS cohort, PMOS was defined as either having a PCOS diagnosis (ICD-10-CM E28.2), or an irregular menstruation (ICD-10-CM N92.6) and hirsutism (ICD-10-CM L68.0) diagnosis, or an irregular menstruation (ICD-10-CM N92.6) and androgen excess (ICD-10-CM E28.1) diagnosis. PMOS controls were defined as having a physical examination (ICD-10-CM Z00.0) and none of the PMOS case criteria. PMOS participants also had to satisfy stringent exclusion criteria to avoid confounders. Exclusion criteria for PMOS consisted of Maternal care for benign tumor of corpus uteri (ICD-10-CM O34.1), Leiomyoma of uterus (ICD-10-CM D25), endometriosis (ICD-10-CM N80), Polyp of corpus uteri (ICD-10-CM N84.0), female pelvic inflammatory disease unspecified (ICD-10-CM N73.9), hypothyroidism (ICD-10-CM E03.8, E03.9), hyperprolactinemia (ICD-10-CM E22.1), and adrenal hyperplasia (ICD-10-CM E27.8, Q89.1). Description of inclusion and exclusion criteria for each cohort can be found in Table 1.

For the PMOS and Pain cohort, PMOS was defined the same as above. PMOS and Pain cases were defined as patients with a PMOS case as well as being diagnosed for either abdominal and pelvic pain (ICD-10-CM R10) or dysmenorrhea (ICD-10-CM N94.6) ± 3months from their first PMOS diagnosis. PMOS and Pain controls were defined as patients with PMOS, but no pain diagnoses. Description of inclusion and exclusion criteria for each cohort can be found in Table 1.

To compare cohorts (cases/controls), the first documented encounter or PMOS (case/controls) or PMOS and Pain (case/controls) was defined as an ‘index event’ in TriNetX. Index events are the specific dates a patient satisfies all selected cohort criteria. Baseline characteristics are all assessed *before* the index event while all health outcomes are assessed *after* the index event.

### Propensity score matching

**Figure 1.**
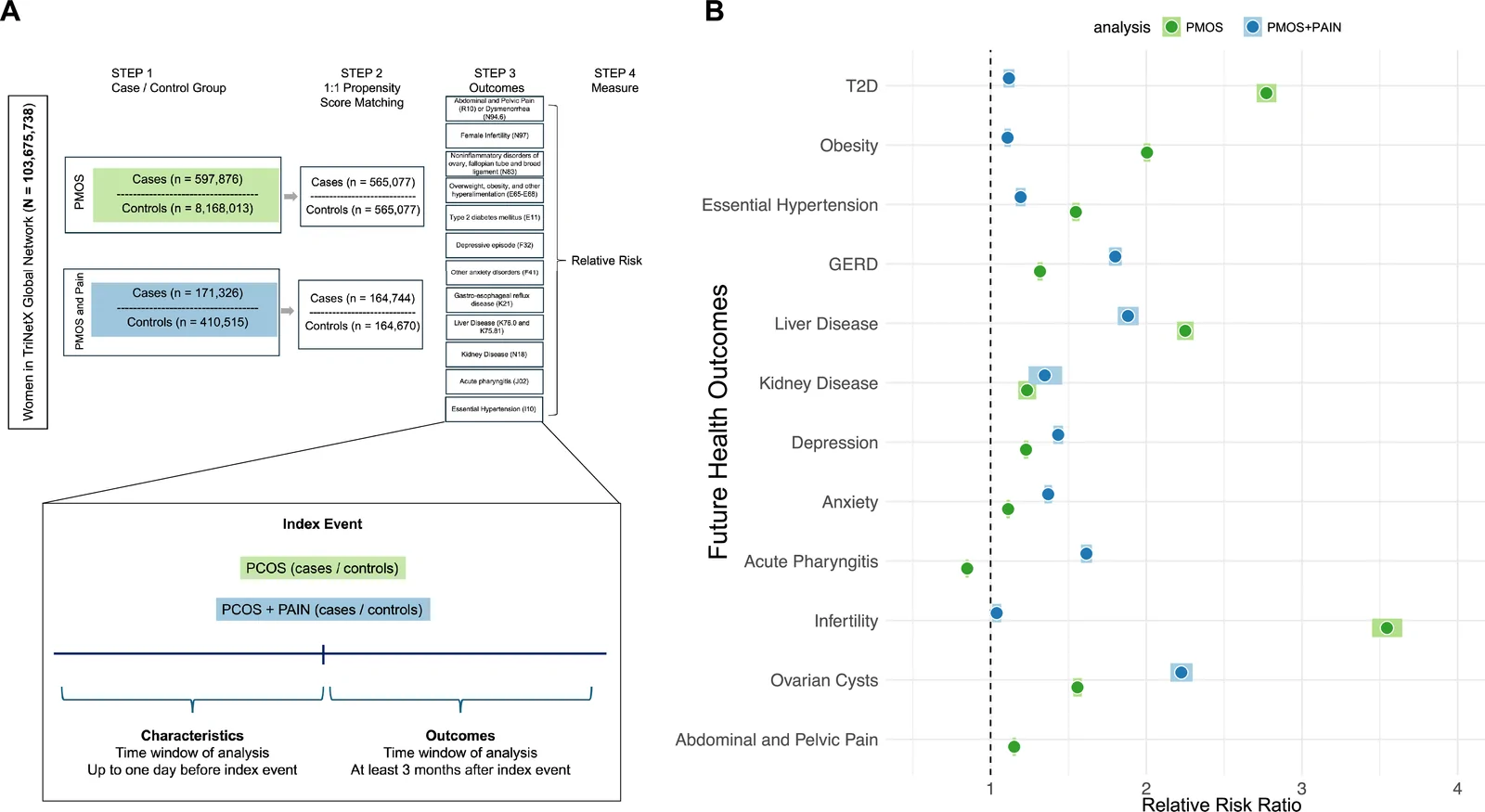
Future health outcomes associated with PMOS and PMOS + Pain. (**A**) Analysis pipeline to calculate relative risk ratios (RR) for future health outcomes in PMOS (green) and PMOS and Pain (blue) cohorts for the 103,675,738 women queried. STEP 1 shows the number of women in the case and controls for both the PMOS (green) and PMOS and Pain (blue) cohorts. STEP 2 shows the number of cases and controls after 1:1 propensity score matching. STEP 3 shows the different future health conditions that were considered for future health outcomes. STEP 4 shows that the final step is calculating the relative risk for the future health outcomes. Popout box is a schematic representing how events were indexed in TriNetX for both the PMOS (green) and PMOS and Pain (blue) cohorts. (**B**) Relative risk ratios (RR) (x-axis) for future health outcomes (y-axis) for both PMOS (green) and PMOS and Pain (blue) cohorts. Darker hued circles indicate RR, while lighter hued boxes indicate the 95% confidence intervals. The black dashed line is set 1 and is the threshold for RR, where >1 is increased RR and <1 is decreased RR.

The TriNetX platform uses a cohort matching method called 1:1 propensity score matching (Austin, 2011). For each cohort analysis, cases were matched on the following criteria: age at the index event, self-reported race, overweight, obesity, and other hyperalimentation (ICD-10-CM E65-E69) status, T2DM (ICD-10-CM E11) status, essential (primary) hypertension (ICD-10-CM I10) status, and hyperlipidemia, unspecified (ICD-10-CM E78.5) status. Baseline conditions were assessed up to one day before the index event. Figure 1A, *top* shows the number of patients in each case and control cohort both at baseline and after propensity score matching. Figure 1A, *bottom* shows a graphical representation of the relationship among index events and outcomes specified in this study. The number of cases and controls before and after matching for the PMOS and PMOS and Pain cohorts can be found in Table 2, Table 3 respectively.

**Table 2.** Demographic results for PMOS case and control cohorts before and after 1:1 propensity score matching. For each cohort analysis, cases were matched on the following criteria: age at the index event, self-reported race, overweight, obesity, and other hyperalimentation (ICD-10-CM E65-E69) status, type 2 diabetes mellitus (T2D) (ICD-10-CM E11) status, essential (primary) hypertension (ICD-10-CM I10) status, and hyperlipidemia, unspecified (ICD-10-CM E78.5) status. Baseline conditions were assessed up to 1 day before the index event.

		Before						After					
Characteristic ID	Characteristic name	PMOS	% PMOS	No PMOS	% No PMOS	p-value	SD	PMOS	% PMOS	No PMOS	% No PMOS	p-value	SD
	Total	576,876		8168013				565,077		565,077			
AI	Age at index	28.13 (±9.04)		43.31 (±20.07)				28.15 (±9.03)		28.16 (±9.04)		2.86E-01	0.00201
1002–5	American Indian or Alaska Native	2843	0.50%	22,878	0.29%	0.00E+00	0.03479	2843	0.50%	1692	0.30%	0.00E+00	0.03222
2028–9	Asian	27,410	4.85%	318,481	3.97%	0.00E+00	0.04296	27,397	4.85%	27,951	4.95%	1.57E-02	0.00454
2054–5	Black or African American	74,376	13.15%	940,271	11.71%	0.00E+00	0.04380	74,335	13.16%	74,972	13.27%	7.68E-02	0.00333
2076–8	Native Hawaiian or Other Pacific Islander	2810	0.50%	22,962	0.29%	0.00E+00	0.03380	2810	0.50%	1704	0.30%	0.00E+00	0.03104
2131–1	Other race	35,155	6.22%	386,380	4.81%	0.00E+00	0.06161	35,070	6.21%	34,717	6.14%	1.68E-01	0.00260
UNK	Unknown race	91,101	16.11%	1,792,170	22.31%	0.00E+00	0.15797	91,100	16.12%	92,810	16.42%	1.31E-05	0.00820
2106–3	White	331,862	58.68%	4,549,060	56.64%	0.00E+00	0.04137	331,522	58.67%	331,231	58.62%	5.78E-01	0.00105
	Other	131,909	23%	2,224,390	28%			131,823	23%	130,923	23%		
2186–5	Not Hispanic or Latino	356,042	62.95%	4,273,952	53.21%	0.00E+00	0.198447	355,745	62.96%	315,837	55.89%	0.00E+00	0.144199
UN	Unknown ethnicity	141,799	25.07%	3,286,982	40.92%	0.00E+00	0.341983	141,761	25.09%	201,808	35.71%	0.00E+00	0.232572
2135–2	Hispanic or Latino	67,716	11.97%	471,268	5.87%	0.00E+00	0.215461	67,571	11.96%	47,432	8.39%	0.00E+00	0.118087
E65-E68	Overweight, obesity, and other hyperalimentation	92,042	16.28%	718,866	8.95%	0.00E+00	0.22199	91,562	16.20%	91,393	16.17%	6.66E-01	0.00081
I10	Essential (primary) hypertension	33,561	5.93%	1,233,003	15.35%	0.00E+00	0.30898	33,554	5.94%	33,531	5.93%	9.27E-01	0.00017
E11	Type 2 diabetes mellitus	19,880	3.52%	442,620	5.51%	0.00E+00	0.09624	19,847	3.51%	20,462	3.62%	1.81E-03	0.00587
E78.5	Hyperlipidemia, unspecified	16,734	2.96%	737,752	9.19%	0.00E+00	0.26295	16,733	2.96%	16,355	2.89%	3.49E-02	0.00397
R10	Abdominal and pelvic pain	100,807	17.82%	1,122,832	13.98%	0.00E+00	0.10530	100,661	17.81%	99,781	17.66%	3.02E-02	0.00408
N94.6	Dysmenorrhea, unspecified	16,107	2.85%	137,898	1.72%	0.00E+00	0.07580	16,070	2.84%	18,870	3.34%	0.00E+00	0.02863
N97	Female infertility	13,244	2.34%	34,447	0.43%	0.00E+00	0.16421	13,241	2.34%	4333	0.77%	0.00E+00	0.12767
N83	Noninflammatory disorders of ovary, fallopian tube and broad ligament	22,160	3.92%	138,266	1.72%	0.00E+00	0.13300	22,144	3.92%	15,098	2.67%	0.00E+00	0.06989
HS200	Contraceptives, systemic	84,147	14.88%	651,819	8.12%	0.00E+00	0.21324	84,076	14.88%	91,954	16.27%	0.00E+00	0.03845
6809	Metformin	38,551	6.82%	260,773	3.25%	0.00E+00	0.16386	38,506	6.81%	14,589	2.58%	0.00E+00	0.20104
9997	Spironolactone	17,819	3.15%	103,247	1.29%	0.00E+00	0.12691	17,813	3.15%	7063	1.25%	0.00E+00	0.12994

**Table 3.** Demographic and baseline characteristics for PMOS and Pain case and control cohorts before and after 1:1 propensity score matching. For each cohort analysis, cases were matched on the following criteria: age at the index event, self-reported race, overweight, obesity, and other hyperalimentation (ICD-10-CM E65-E69) status, type 2 diabetes mellitus (T2D) (ICD-10-CM E11) status, essential (primary) hypertension (ICD-10-CM I10) status, and hyperlipidemia, unspecified (ICD-10-CM E78.5) status. Baseline conditions were assessed up to 1 day before the index event.

		Before						After					
Characteristic ID	Characteristic name	PMOS +Pain	% PMOS +Pain	PMOS - Pain	% PMOS - Pain	p-value	SD	PMOS +Pain	% PMOS +Pain	PMOS - Pain	% PMOS - Pain	p-value	SD
	Total	171,326		410,515				164,744		164,744			
AI	Age at index	28.53 (±8.78)		28.41 (±9.20)	100%	3.08E-06	0.0136	28.39 (±8.81)		28.40 (±9.03)	100%	8.48E-01	0.00067
1002–5	American Indian or Alaska Native	950	0.56%	1931	0.48%	1.34E-04	0.0109	933	0.57%	740	0.45%	2.24E-06	0.01649
2028–9	Asian	6075	3.58%	21,499	5.36%	0.00E+00	0.0862	6044	3.67%	5969	3.63%	4.86E-01	0.00243
2054–5	Black or African American	24,692	14.55%	50,827	12.67%	0.00E+00	0.0548	23,657	14.37%	25,050	15.21%	8.05E-12	0.02383
2076–8	Native Hawaiian or Other Pacific Islander	937	0.55%	1927	0.48%	4.57E-04	0.0100	928	0.56%	756	0.46%	2.64E-05	0.01465
2131–1	Other race	10,935	6.44%	24,595	6.13%	8.34E-06	0.0128	10,637	6.46%	10,595	6.43%	7.66E-01	0.00104
UNK	Unknown race	22,174	13.06%	69,034	17.21%	0.00E+00	0.1158	22,043	13.39%	22,107	13.43%	7.43E-01	0.00114
2106–3	White	104,003	61.26%	231,439	57.68%	0.00E+00	0.0730	100,428	60.99%	99,453	60.40%	5.04E-04	0.01212
	Other	34,996	21%	97,487	24%			34,541	21%	34,198	21%		
2186–5	Not Hispanic or Latino	109,689	64.59%	250,682	62.40%	0	0.045	105,912	64.29%	106,378	64.57%	0.08991917	0.0059
UN	Unknown ethnicity	36,480	21.48%	106,152	26.43%	0	0.116	35,807	21.74%	38,637	23.45%	4.4381E-32	0.0411
2135–2	Hispanic or Latino	23,662	13.93%	44,881	11.17%	0	0.083	23,025	13.98%	19,729	11.98%	0	0.0596
E65-E68	Overweight, obesity, and other hyperalimentation	57,508	33.88%	58,705	14.63%	0.00E+00	0.4608	52,443	31.85%	52,512	31.89%	7.96E-01	0.00090
I10	Essential (primary) hypertension	21,094	12.43%	20,962	5.22%	0.00E+00	0.2559	17,411	10.57%	17,868	10.85%	1.00E-02	0.00897
E11	Type 2 diabetes mellitus	13,316	7.84%	12,421	3.10%	0.00E+00	0.2100	10,826	6.57%	10,529	6.39%	3.56E-02	0.00732
E78.5	Hyperlipidemia, unspecified	12,548	7.39%	10,765	2.68%	0.00E+00	0.2165	9994	6.07%	9648	5.86%	1.09E-02	0.00887
R10	Abdominal and pelvic pain	73,588	43.35%	38,148	9.51%	0.00E+00	0.8310	71,136	43.20%	20,710	12.58%	0.00E+00	0.72651
N94.6	Dysmenorrhea, unspecified	12,010	7.07%	5391	1.34%	0.00E+00	0.2884	11,704	7.11%	2977	1.81%	0.00E+00	0.25894
N97	Female infertility	10,047	5.92%	9443	2.35%	0.00E+00	0.1798	9689	5.88%	4305	2.61%	0.00E+00	0.16263
N83	Noninflammatory disorders of ovary, fallopian tube, and broad ligament	18,080	10.65%	9851	2.46%	0.00E+00	0.3358	17,401	10.57%	5146	3.13%	0.00E+00	0.29795
HS200	Contraceptives, systemic	50,158	29.55%	54,520	13.59%	0.00E+00	0.3955	48,365	29.37%	26,568	16.13%	0.00E+00	0.31975
6809	Metformin	29,039	17.11%	26,583	6.63%	0.00E+00	0.3284	26,679	16.20%	14735	8.95%	0.00E+00	0.22008
9997	Spironolactone	12,536	7.38%	12,620	3.15%	0.00E+00	0.1907	11,780	7.15%	5889	3.58%	0.00E+00	0.15927

### Future health outcomes

For each of the cohorts listed above, we calculated relative risk ratios (RR) with 95% confidence intervals for 11 future health outcomes: abdominal and pelvic pain (R10) or dysmenorrhea (N94.6), female infertility (ICD-10-CM N97), noninflammatory disorders of ovary, fallopian tube, and broad ligament (ICD-10-CM N83), obesity (ICD-10-CM E65-E68), T2D (ICD-10-CM E11), depressive episode (ICD-10-CM F32), other anxiety disorders (F41), gastroesophageal reflux disease (GERD; ICD-10-CM K21), nonalcoholic steatohepatitis (ICD-10-CM K75.81) or fatty liver, not elsewhere classified (ICD-10-CM K76.0), chronic kidney disease (ICD-10-CM N18), and essential hypertension (ICD-10-CM I10). The RR for future health outcomes was calculated on participants who satisfied the 1:1 propensity score matching criteria (above). Differences in relative risks were calculated for significance by calculating the difference of two estimates (Altman and Bland, 2003). Future health outcomes were only considered if their first occurrence was at least 3 months after the index event. Figure 1A shows the health outcomes assessed after 1:1 propensity score matching (Austin, 2011; Guo and Fraser, 2014; Haukoos and Lewis, 2015). These analyses were done within the TriNetX platform, and no individual-level data was extracted from the platform.

### Self-reported race stratified sub-analysis

We did a follow-up analysis looking at health outcomes for patients with PMOS and PMOS and Pain compared to matched controls stratified by self-reported race and ethnicity. The following race categories are present in the TriNetX platform: American Indian or Alaskan Native, Asian, Black or African American, Native Hawaiian or Other Pacific Islander, Other, White, and Unknown Race. For our analysis, we included the following four race categories: Asian, Black or African American, and Other (American Indian or Alaskan Native or Native Hawaiian or Other Pacific Islander, or Other), and White. Due to the small population sizes of American Indian or Alaskan Native or Native Hawaiian or Other Pacific Islander, or Other, we decided to combine these four self-reported race groups together into one ‘Other’ population. For the PMOS and Pain cohorts, we looked at ovarian cysts, infertility, obesity, T2D, depression, anxiety, GERD, pharyngitis, essential hypertension, liver disease, and kidney disease stratified by self-reported race. Figure 3A shows the self-reported race sub-analysis workflow.

### PMOS medication sub-analysis

We performed a follow-up analysis looking at the number of patients with PMOS who were documented as having pain before being prescribed three common PMOS medications (systemic contraceptives [VA: HS200], metformin [RxNorm 6809], or spironolactone [RxNorm 9997]) as shown in Figure 4A. Three cohorts were created on January 16, 2026, in the TriNetX Global Network. There were approximately 190,888,407 million patients from 170 healthcare organizations in 19 different countries. Patients were included if they had a PMOS diagnosis (described above) *and* (1) ever had a systemic contraceptives prescription but not a metformin or spironolactone prescription, (2) ever had a metformin prescription but not a systemic contraceptives or spironolactone prescription, and (3) ever had a spironolactone but not a systemic contraceptives or metformin prescription. For patients with PMOS, we counted the number of participants who had a diagnosis code for either dysmenorrhea or abdominal and pelvic pain before the index event. The index event was defined as a participant having a PMOS diagnosis and a medication prescription at the same time. We then counted the number of patients with PMOS who reported either dysmenorrhea or abdominal and pelvic pain after the index event. We further compared the change in prevalence before and after the indexed event.

## Results

### Demographics of women with PMOS in the TriNetX global network

**Figure 2.**
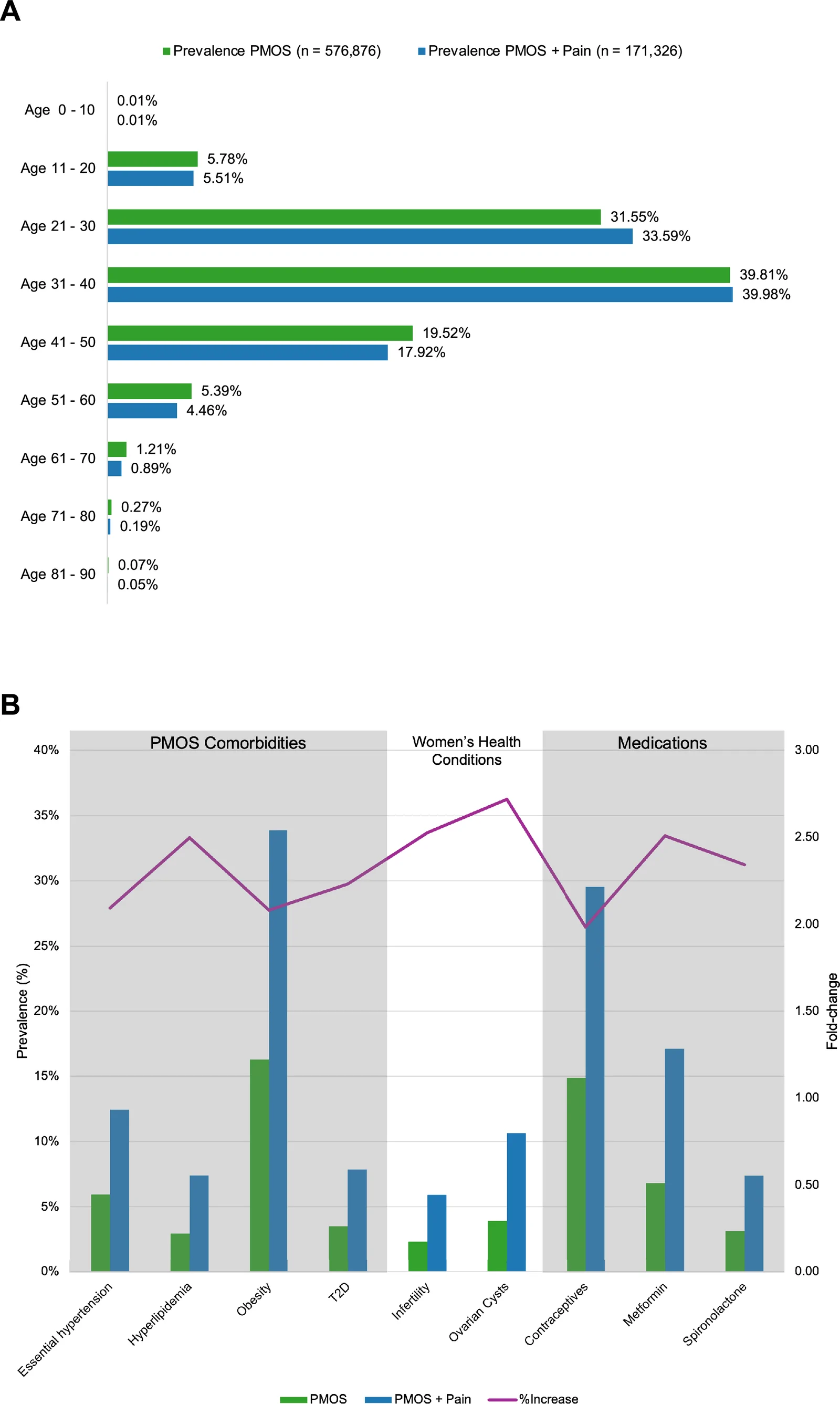
Prevalence of PMOS, PMOS and Pain, and associated conditions. (**A**) Bar plots show the prevalence (%) of overall PMOS (green) and PMOS and Pain (blue) stratified by 10-year age groups. The total number of women with PMOS is 576,876 and the total number of women with PMOS and Pain is 171,326. (**B**) Bar plots show the prevalence (%) (left y-axis) of different diseases associated with PMOS (green) and PMOS and Pain (blue) (x-axis). The purple line indicates the prevalence fold-change between the PMOS and PMOS and Pain cohorts (right y-axis).

We first identified participants with PMOS and associated comorbidities. The demographics and characteristics of both the PMOS and non-PMOS cohorts are detailed in Table 2. We queried the 103,675,738 women of any age from 172 HCOs in the TriNetX Global Network for PMOS and associated comorbidities. After applying stringent inclusion/exclusion criteria for PMOS and subsequent controls (see Methods), we found a 5.6% (n=576,077) prevalence of PMOS at an average age of 28.1 (SD ± 9.04) in this population. When we stratified the PMOS participants in 10-year age groups, we observed that the majority of PMOS patients are either 21–30 years old (30.46%) or 31–40 years old (38.42%) Figure 2A. Of those participants with PMOS, 4.85%, 13.15%, 23.32%, and 58.68% self-identified as Asian, Black or African American, Other, or White, respectively (Table 2).

We then examined the prevalence of PMOS-associated comorbidities represented in the PMOS cohort. We observed that 16.28% of patients with PMOS had a diagnosis code for obesity, 5.93% had a diagnosis code for essential hypertension, 3.51% had a diagnosis code for T2D, and 2.96% had a diagnosis code for hyperlipidemia (Table 2). We also investigated the prevalence of other women’s health conditions related to PMOS, such as infertility and ovarian cysts. Table 2 shows that women with PMOS had a 2.34% prevalence of infertility and a 3.92% prevalence of ovarian cysts.

Since many women with PMOS are prescribed medications to help manage symptoms associated with the condition, we aimed to gain a deeper understanding of the prevalence of PMOS-prescribed medications in the PMOS cohort. We found that 14.88% of the PMOS cohort were prescribed systemic oral contraceptives, 6.82% were prescribed metformin, and 3.15% were prescribed spironolactone (Table 2).

A focus of our paper is understanding the impact pain has on women with PMOS; therefore, we looked at the prevalence of both dysmenorrhea and abdominal and pelvic pain. We observed that overall, there was a 20.67% prevalence of pain (2.85% prevalence of dysmenorrhea, 17.82% prevalence of abdominal or pelvic pain) (Table 2).

### Demographics of women with PMOS and Pain in the TriNetX global network

As noted above, 20.67% of women with PMOS also had a pain diagnosis, encompassing either dysmenorrhea or pelvic and abdominal pain. We first examined the demographics of the study participants using data from the TriNetX Global Network. Table 3 shows the comprehensive demographic results, highlighting the distribution of pain diagnoses for age, race, and other relevant demographic factors. This table provides a clear view of the demographic characteristics and their potential influence on the prevalence of pain among individuals with PMOS. Similar to the PMOS cohort, participants with PMOS and Pain had an average age of 28.53 (SD ± 8.78), with the majority of participants being between 21 and 30 years old (31.55%) or 31–40 years old (39.81%). Interestingly, the prevalence of PMOS and Pain was slightly higher at these ages compared to the prevalence of PMOS alone (Figure 2A). Among the women with PMOS and Pain, 3.58% women were Asian, 14.55% Black or African American, 21% categorized as Other, and 61.26% self-reported as White (Table 3).

However, when we looked at the prevalence of PMOS and Pain compared to controls (PMOS without pain) within a self-reported race group, we observed that the highest prevalence of PMOS and Pain was 32.70% in the Black or African American population, followed by 31% in the White population, 26.42% in the Other population, and 22.03% in the Asian population. (Table 4). With respect to PMOS comorbidities, the cohort of individuals with both PMOS and Pain exhibited a higher prevalence of comorbid conditions compared to the entire population of individuals with PMOS. Specifically, 33.88% of PMOS and Pain participants had an obesity diagnosis, 12.43% had an essential hypertension diagnosis, 7.84% had a T2D diagnosis, and 7.39% had a hyperlipidemia diagnosis. These high prevalences represent a respective increase of 16.28%, 5.93%, 3.52%, and 2.96% compared to all participants with PMOS. Notably, as illustrated in Figure 2B, there is at least a two-fold increase in the prevalence of each comorbid condition among those with both PMOS and Pain. This substantial increase highlights the heightened risk and burden of comorbidities within the PMOS and Pain cohort.

**Table 4.** Counts and prevalence (%) of PMOS and Pain cases and controls (PMOS without Pain) for self-reported race groups.

Self-reported race group	PMOS + Pain	PMOS - Pain	Prevalence (%)
Asian	6,075	21,499	22.03
Black or African American	24,692	50,827	32.70
Other	34,996	97,487	26.42
White	104,003	231,439	31.00

We observed a similar trend with respect to two diseases affecting women with PMOS. We see a twofold increase in the prevalence of infertility (5.92%) and ovarian cysts (10.65%) in the PMOS and Pain cohort when compared to the PMOS cohort, as shown in Figure 2B. Further, there is at least a twofold increase in prescriptions for all three common PMOS symptom-management medications in the PMOS and Pain cohort compared to the PMOS cohort (Figure 2B).

### Risk of future health outcomes for all PMOS vs. PMOS and Pain cohorts

Given that PMOS symptoms first manifest in puberty and during reproductive years, we aimed to assess the risk for patients with PMOS, and those with both PMOS and Pain, developing future health outcomes. The risk (%) for the future health outcomes assessed in this study can be found in the risk column of Table 5. To start, we see that 21% of women with PMOS overall were at risk for a future diagnosis of Pain (abdominal and pelvic pain or dysmenorrhea). Of the comorbidities of PMOS, obesity, T2D, and essential hypertension had 20.7%, 5.1%, and 7.7% increased risk in the PMOS overall cohort and 20.4%, 5.2%, and 8.2% increased risk in the PMOS and Pain cohort respectively. Liver disease and kidney disease, which are on the rise in PMOS patients, were found to have a 4.0% and 0.7% increased risk in the PMOS cohort and at 5.4% and 0.9% increased risk in the PMOS and Pain cohort. The ‘Explore Outcome’ feature on the TriNetX platform revealed that anxiety, depression, gastroesophageal reflux disease (GERD), and acute pharyngitis were the most common future health outcomes for women diagnosed with PMOS and Pain. When we looked at risk for these future health outcomes, we observed that 17.1%, 11.5%, 10.5%, and 10.0% of patients with PMOS and 20.1%, 13.7%, 13.5%, and 13.3% of patients with PMOS and Pain were at risk of developing anxiety, depression, acute pharyngitis, and GERD respectively. Overall, besides obesity, patients with PMOS and Pain showed a higher risk for the investigated future health outcomes than PMOS alone. We explore the relationship of future health outcome risk further in the results below.

**Table 5.** Relative risk ratios (RR) for PMOS and PMOS and Pain cases and control cohorts. Significant differences in RR between PMOS and PMOS and Pain cohorts are bolded.

Outcomes	Cohort name	Patients in cohort	Patients with outcome	Risk (%)	Relative risk ratio (RR)	95% CI (lower)	95% CI (upper)	p-Value
Abdominal and pelvic pain	PMOS + pain	0	0		NA	NA	NA	NA
Abdominal and pelvic pain	PMOS - pain	141,399	13,599	9.6				
Abdominal and pelvic pain	PMOS overall	412,548	86,597	21.0	1.15	1.14	1.16	
Abdominal and pelvic pain	PMOS controls	434,896	79,269	18.2				
Acute pharyngitis	PMOS + pain	129,728	17,522	13.5	1.62	1.58	1.65	**1.64e-397**
Acute pharyngitis	PMOS - pain	144,688	12,096	8.4				
Acute pharyngitis	PMOS overall	499,241	52,252	10.5	0.85	0.84	0.86	
Acute pharyngitis	PMOS controls	478,551	58,938	12.3				
Anxiety	PMOS + pain	110,687	22,193	20.1	1.37	1.35	1.39	**2.33E-11**
Anxiety	PMOS - pain	127,316	18,631	14.6				
Anxiety	PMOS overall	446,953	76,641	17.1	1.11	1.10	1.12	
Anxiety	PMOS controls	446,119	68,745	15.4				
Depression	PMOS + pain	124,618	17,015	13.7	1.43	1.40	1.47	**1.72E-05**
Depression	PMOS - pain	137,978	13,136	9.5				
Depression	PMOS overall	480,632	55,085	11.5	1.23	1.21	1.24	
Depression	PMOS controls	487,289	45,485	9.3				
Essential hypertension	PMOS + pain	143,081	11,675	8.2	1.19	1.16	1.22	**4.96E-56**
Essential hypertension	PMOS - pain	140,519	9611	6.8				
Essential hypertension	PMOS overall	507,320	39,064	7.7	1.55	1.52	1.57	
Essential hypertension	PMOS controls	522,019	25,971	5.0				
GERD	PMOS + pain	130,549	17,386	13.3	1.80	1.76	1.84	**2.52E-48**
GERD	PMOS - pain	147,029	10,873	7.4				
GERD	PMOS overall	506,269	50,438	10.0	1.32	1.30	1.33	
GERD	PMOS controls	516,304	39,041	7.6				
Infertility	PMOS + pain	164,744	9358	5.7	1.04	1.01	1.07	**6.04e-820**
Infertility	PMOS - pain	164,744	9005	5.5				
Infertility	PMOS overall	530,186	22,014	4.2	3.55	3.45	3.64	
Infertility	PMOS controls	559,224	6548	1.2				
Kidney disease	PMOS + pain	163,279	1409	0.9	1.35	1.24	1.46	6.64E-02
Kidney disease	PMOS - pain	163,529	1047	0.6				
Kidney disease	PMOS overall	561,790	3884	0.7	1.23	1.18	1.29	
Kidney disease	PMOS controls	561,941	3148	0.6				
Liver disease	PMOS + pain	153,385	8287	5.4	1.88	1.82	1.95	**1.35E-23**
Liver disease	PMOS - pain	159,449	4576	2.9				
Liver disease	PMOS overall	548,043	21,676	4.0	2.25	2.20	2.30	
Liver disease	PMOS controls	557,915	9806	1.8				
Obesity	PMOS + pain	99,165	20,223	20.4	1.11	1.09	1.13	**2.07e-506**
Obesity	PMOS - pain	92,475	17,003	18.4				
Obesity	PMOS overall	391,393	80,997	20.7	2.00	1.98	2.03	
Obesity	PMOS controls	444,137	45,849	10.3				
Ovarian cysts	PMOS +pain	138,617	10,601	7.6	2.23	2.16	2.30	**2.24E-36**
Ovarian cysts	PMOS - pain	156,505	5379	3.4				
Ovarian cysts	PMOS overall	527,152	29,047	5.5	1.56	1.53	1.59	
Ovarian cysts	PMOS controls	547,043	19,347	3.5				
T2D	PMOS +Pain	150,919	7882	5.2	1.12	1.08	1.15	**4.28e-453**
T2D	PMOS - Pain	149,586	6989	4.7				
T2D	PMOS Overall	528,769	27,124	5.1	2.77	2.71	2.84	
T2D	PMOS Controls	541,299	10,018	1.9				

### Relative risk of future health outcomes for all PMOS vs. PMOS and Pain

We next calculated the RR for matched PMOS patient cohorts with their respective controls and PMOS and Pain patient cohorts with their respective controls (see Methods). Results are visualized in Figure 1B and cohort counts, RRs, and p-values for differences in risk ratios are provided in Table 5.

In Figure 1B, we see the RR for both participants within the PMOS cohort (green) for 12 health outcomes and PMOS and Pain cohort (blue) for 11 health outcomes. Aside from acute pharyngitis, the PMOS cohort has significantly increased risk for developing the following future health outcomes compared to matched controls: T2D, obesity, essential hypertension, GERD, liver disease, kidney disease, depression, anxiety, infertility, ovarian cysts, and pain. The PMOS and Pain cohort meanwhile has significantly increased risk for developing all of the following future health outcomes compared to their matched controls: T2D, obesity, essential hypertension, GERD, kidney disease, depression, anxiety, acute pharyngitis, infertility, ovarian cysts, and pain.

While almost all of the RR are increased for case cohorts compared to match controls, a few results stand out as being particularly interesting. Infertility, for example, a common complication associated with PMOS, has a RR of 3.55 (95% CI 3.45–3.64) in PMOS cases overall. Meanwhile, the RR for a future outcome of infertility for women with PMOS and Pain is a near-insignificant 1.04 (95% CI 1.04–1.07). This difference in relative risks has a p-value of 6.04E-820 (Table 5). On the other hand, ovarian cysts have a RR of 1.56 (95% CI 1.53–1.59) in PMOS cases overall, but an even higher RR in PMOS and Pain cases (RR = 2.23, 95% CI 2.16–2.30). This relative risk difference is also statistically significant (p-value = 2.24E-36; Table 5). In GERD, depression, anxiety, and acute pharyngitis all had higher RRs in PMOS and Pain cases vs. controls compared to PMOS cohort cases compared to controls (Figure 1B, Table 5). We see that GERD, acute pharyngitis, depression, and anxiety are 1.80 (95% CI 1.76–1.84), 1.62 (95% CI 1.58–1.65), 1.43 (95% CI 1.40–1.47), and 1.37 (95% CI 1.35–1.39) in the PMOS and Pain cohort compared to 1.32 (95% CI 1.30–1.33), 0.85 (95% CI 0.84–0.86), 1.23 (95% CI 1.21–1.24), and 1.11 (95% CI 1.10–1.12) in the entire PMOS cohort at statistical significance (Figure 1B, Table 5).

PMOS and PMOS and Pain cohorts were at comparable increased risk for developing future kidney disease. The RR of the PMOS cohort was 1.23 (95% CI 1.18–1.29) while the RR for the PMOS and Pain cohort was 1.35 (95% CI 1.24–1.46; Figure 1B). The relative risk difference was not statistically significant (p-value = 6.64E-02; Table 5).

Obesity, T2D, essential hypertension, and liver disease had increased risk for the PMOS cohort compared to the PMOS and Pain cohort. T2D had a RR of 2.77 (95% CI 2.71–2.84) in the PMOS cohort compared to 1.12 (95% CI 1.08–1.15) in the PMOS and Pain cohort. Obesity had a RR of 2.00 (95% CI 1.98–2.03) in the PMOS cohort compared to 1.11 (95% CI 1.09–1.13) in the PMOS and Pain cohort. Meanwhile, the RR for liver disease was 2.25 (95% CI 2.20–2.30) in the PMOS cohort compared to 1.89 (95% CI 1.82–1.95) in the PMOS and Pain cohort. Finally, essential hypertension had a RR of 1.55 (95% CI 1.52–1.57) in the PMOS cohort and 1.19 (95% CI 1.16–1.22) in the PMOS and Pain cohort (Figure 1B). All relative risk differences were statistically significant (Table 5).

### Relative risk of future health outcomes stratified by self-reported race

**Figure 3.**
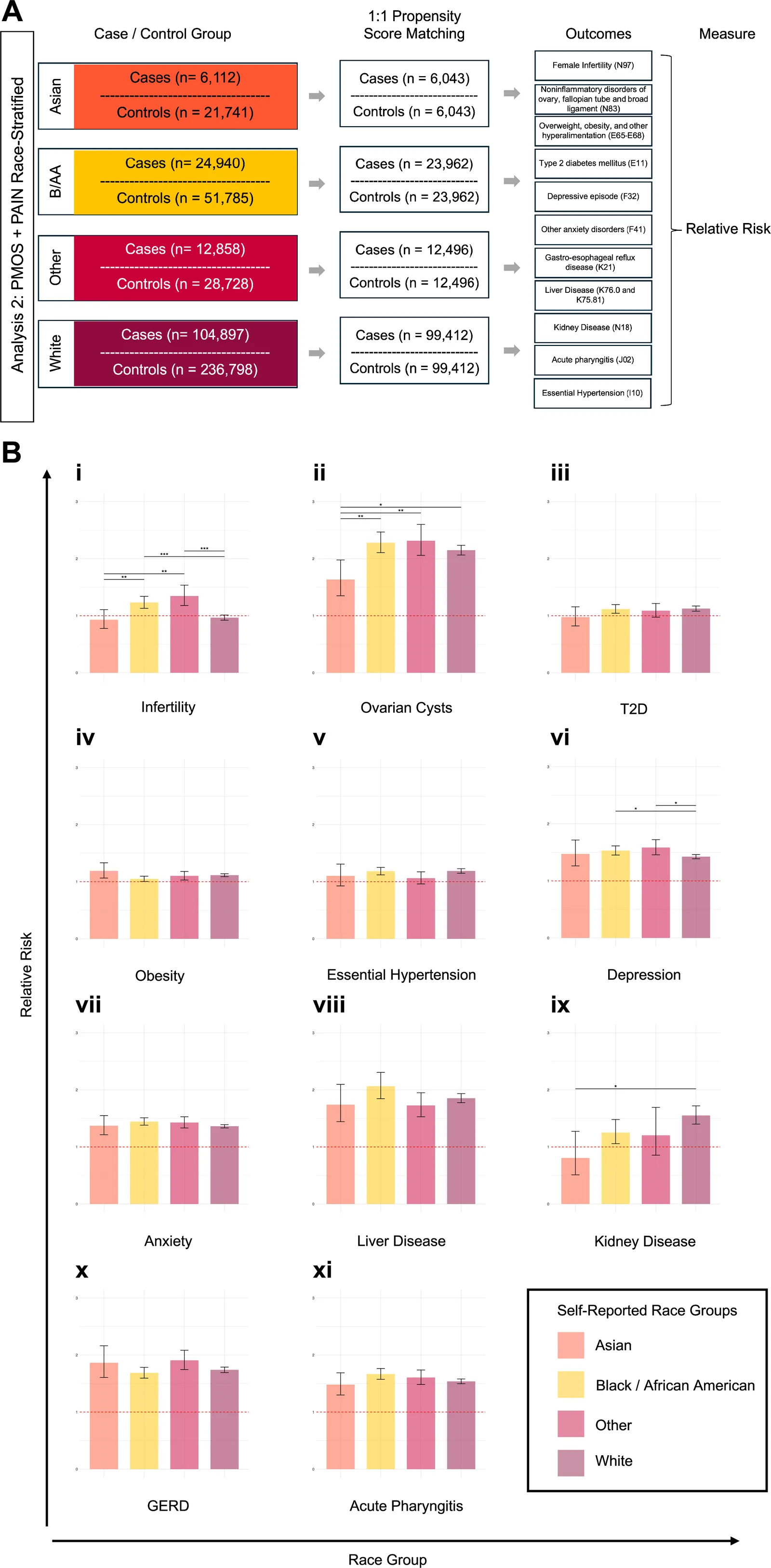
Self-reported race-stratified relative risk ratios for future health outcomes. (**A**) Self-reported race-stratified TriNetX relative risk ratio analysis pipeline for future health outcomes in PMOS and Pain cohorts. Colors represent different self-reported race groups: Asian (orange), Black or African American (yellow), Other (red), White (purple). (**B**) Relative risk ratios (RR) for future health outcomes (y-axis) stratified by self-reported race. Colors represent different self-reported race groups (x-axis): Asian (orange), Black or African American (yellow), Other (red), White (purple). Error bars represent the 95% confidence intervals. Significant differences between RR are represented by asterisks (*), where p-value ≤0.05 = *, p-value ≤0.005 = **, p-value ≤0.0005 = ***, and -value ≤0.00005 = ****. Red dashed line is set 1 and is the threshold for RR, where >1 is increased RR and <1 is decreased RR. Figure 3—source data 1.This file contains the relative risk ratios (RR) and 95% confidence intervals for the race-stratified PMOS and Pain cohort compared to controls. Figure 3—source data 2.This file contains the signficance values for future health RR differences between each self-reported race group combination with PMOS and Pain cohort compared to controls.

**Figure 4.**
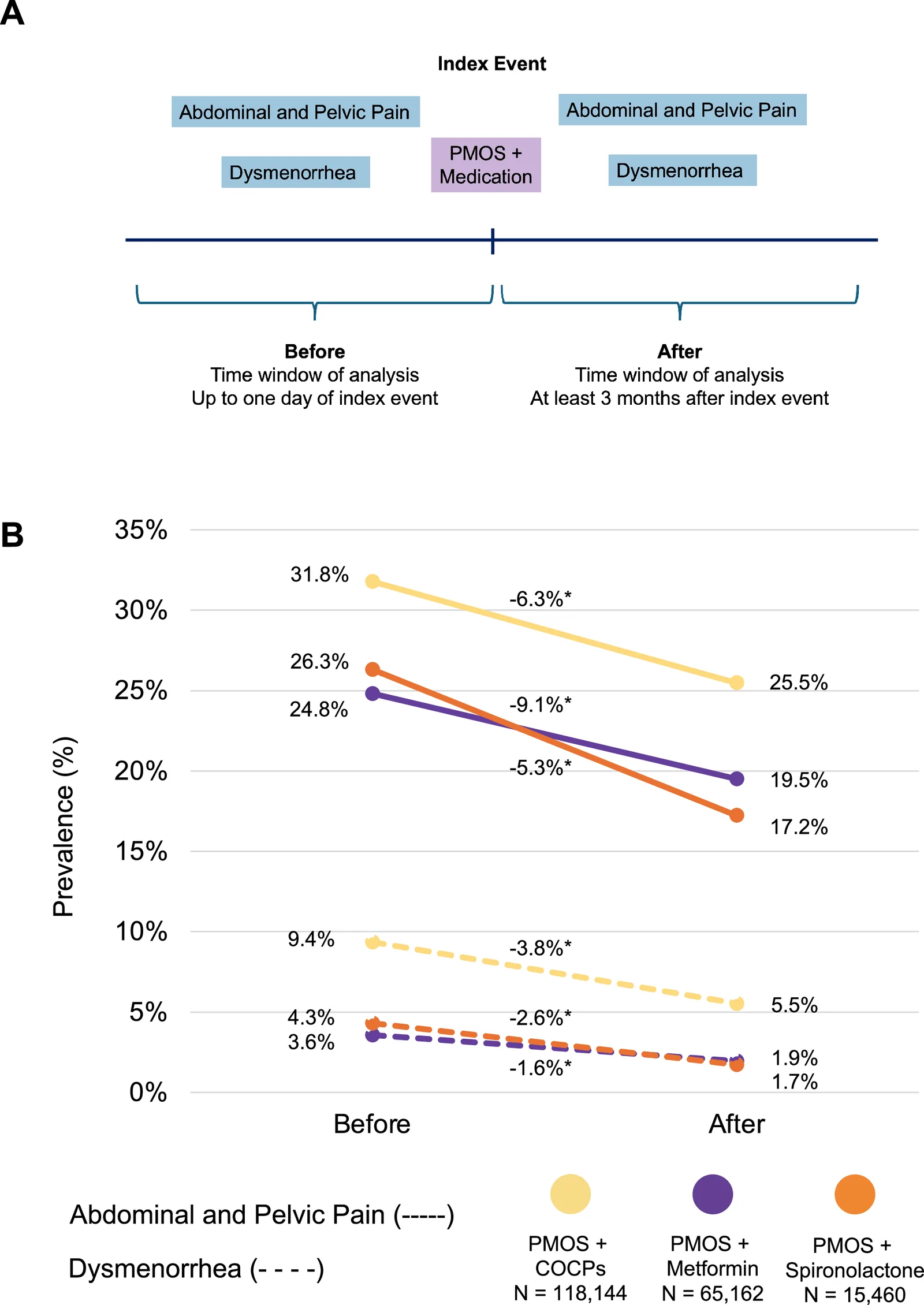
Prevalence of Pain for women with PMOS before and after medications. (**A**) Schematic representing how the PMOS and medication events were indexed in TriNetX for both abdominal and pelvic pain and dysmenorrhea. (**B**) Prevalence (%) changes (y-axis) of pain for women with PMOS cohort before and after prescription of COCPs (yellow), metformin (purple), and spironolactone (orange) (x-axis). Analysis was done separately for abdominal and pelvic pain (solid lines) and dysmenorrhea (dashed lines).

Since we were interested in the impact of Pain for women with PMOS, we next investigated if there were any race-specific risks (self-reported from EHR) for these outcomes. We stratified the PMOS and Pain case and control cohorts by self-reported race and calculated RR for the same 11 future health outcomes (methods) discussed above. Figure 3B(i-xi) shows the RR for each of the 11 future health outcomes in the Asian (orange), Black or African American (yellow), Other (red), and White (purple) PMOS and Pain cohorts. We observed significant race-specific differences in RR for a number of future health outcomes. Figure 3i shows that infertility has increased RR in the Other cohort (RR = 1.35, 95% CI 1.18–1.54) and Black or African American (RR = 1.23, 95% CI 1.13–1.34). Both the Other and Black or African American PMOS and Pain cohorts had significantly increased risk when compared to the Asian and White cohorts (adjusted p-value ≤0.005; Figure 3i, Figure 3—source data 1 and 2). Ovarian cysts had increased RR across Asian (RR = 1.63, 95% CI 1.35–1.98), Black or African American (RR = 2.28, 95% CI 2.11–2.47), Other (RR = 2.31, 95% CI 2.06–2.60), and White (RR = 2.15, 95% CI 2.06–2.23) PMOS and Pain cohorts. However, there was a significantly increased RR for ovarian cysts in the Other, Black or African American, and White PMOS and Pain cohorts compared to the Asian PMOS and Pain cohort (adjusted p-value ≤0.05; Figure 3ii). Meanwhile, while all self-reported race cohorts show at least a 1.42 increased RR for depression, there was a significantly increased RR in Black or African American and Other PMOS and Pain cohorts compared to White PMOS and Pain cohorts (adjusted p-value ≤0.05; Figure 3vi). Interestingly, the Asian PMOS and Pain cohort had a decreased RR (RR = 0.8, 95% CI 0.51–1.27) for kidney disease compared to an increased RR in the Black or African American (RR = 1.53, 95% CI 1.45–1.61), Other (RR = 1.58, 95% CI 1.39–1.46), and White (RR = 1.42, 95% CI 1.39–1.46) PMOS and Pain cohorts (Figure 3ix). Moreover, there was a significant increased RR for kidney disease in the White PMOS and Pain cohort compared to the Asian PMOS and Pain cohort (adjusted p-value ≤0.05; Figure 3ix). None of the PMOS and Pain self-reported race cohorts showed notable RR for the future health outcomes of T2D, Obesity, or Essential Hypertension (Figure 3iii–v). On the other hand, all PMOS and Pain self-reported race groups had increased RR for anxiety (RR at least 1.36, 95% CI 1.33–1.39), GERD (RR at least 1.69, 95% CI 1.59–1.78), and acute pharyngitis (RR at least 1.48, 95% CI 1.30–1.69; Figure 4vi, x and xi).

### Medications prescribed to patients with PMOS may modify pain prevalence

Since women with PMOS are often prescribed medications to help their PMOS symptoms, we aimed to investigate if there were any changes in the pain diagnoses after being prescribed systemic contraceptives (COCPs), metformin, or spironolactone. For patients with PMOS who were prescribed each of the three medications exclusively, we calculated the percent who reported dysmenorrhea or abdominal and pelvic pain both before and after the prescription (see Methods). We found that there were 118,144 women with PMOS who were prescribed systemic contraceptives, 65,162 prescribed metformin, and 15,460 prescribed spironolactone. The prevalence of abdominal and pelvic pain diagnosis was 6–8 x greater than that of a dysmenorrhea diagnosis for PMOS participants before they were prescribed PMOS-related medications (Figure 4B).

Oftentimes, women with PMOS are prescribed COCPs, metformin, and spironolactone to manage their symptoms. We observed that women with PMOS had prescriptions for COCPs 17.8% of the time, metformin 10.3% of the time, and spironolactone 2.3% of the time. As can be observed in Figure 4B, participants with PMOS reported abdominal and pelvic pain at a prevalence of 31.8%, 24.8%, and 26.3% before their first prescription of COCPs, metformin, and spironolactone, respectively. At least 3 months after being prescribed a PMOS-associated medication, we observe a significant reduction in the prevalence of abdominal and pelvic pain. Spironolactone shows the largest reduction of pain prevalence with a –9.1% reduction of pain diagnosis after prescription compared to before, followed by COCPs (–6.3%) and metformin (–5.3%). Similar results are observed for dysmenorrhea. While lower overall, there was also a decreased prevalence of pain for all three medications; the prevalence for dysmenorrhea was 9.4%, 3.6%, and 4.3% for participants with PMOS prescribed COCPs, metformin, and spironolactone, respectively. Unlike with abdominal and pelvic pain, COCP prescriptions were associated with the largest decrease in dysmenorrhea prevalence (–3.8%), followed by spironolactone (–2.6%), and (–1.6%).

## Discussion

Polyendocrine metabolic ovarian syndrome is the most prevalent endocrine disorder among women (Mousa and Tay, 2023; Walters et al., 2018). Diagnosis and treatment plans are customized based on the symptoms presented by women. However, an important yet often overlooked variable is pain, which may manifest as dysmenorrhea, abdominal, or pelvic pain. The use of EHR data has facilitated access to patient records containing longitudinal clinical information, utilizing the readily available International Classification of Diseases (ICD) codes (Wu et al., 2017). Our study aimed to elucidate the prevalence and impact of pain among individuals with PMOS, as well as to investigate the relative risk of future health outcomes and the effectiveness of commonly prescribed medications on pain. Firstly, we observed a significantly higher prevalence of pain among women with PMOS compared to those without the condition. Specifically, 20.67% of women with PMOS reported experiencing pain, compared to 15.7% in the non-PMOS cohort. This increased prevalence reveals the substantial burden of pain as a symptom of PMOS, which often goes underreported and undertreated. Our demographic analysis of women with PMOS and Pain also revealed a difference in diagnosis of pain across self-reported race groups and was especially high in the Black or African American population (32.7%) and White population (30.78%). These findings suggest that pain is a significant symptom of PMOS that can vary across different demographic groups. The high prevalence of pain underscores the need for healthcare clinicians to routinely assess and address pain in the management of PMOS, particularly in racially diverse populations. The diversity in pain perception and reporting among different racial groups can be influenced by a variety of factors, including genetic differences, cultural attitudes towards pain, access to healthcare, and socioeconomic status. Women of different racial groups often experience different severities in pain (Portenoy et al., 2004). This can lead to disparities in pain management and treatment outcomes (Campbell and Edwards, 2012; Jamieson and Steege, 1996). Additionally, cultural differences may also affect how individuals report pain and their willingness to seek medical help (Hadjiconstantinou et al., 2017).

PMOS manifests with many other concomitant conditions (Anagnostis et al., 2018; Asuncion et al., 2000; Balen et al., 2016; Escobar-Morreale et al., 2011; Hadjiconstantinou et al., 2017; Kitzinger and Willmott, 2002; Patel, 2018). Our study revealed that women with PMOS and Pain have at least a twofold increased prevalence of other health conditions at baseline compared to women with PMOS in general. The prevalence of obesity in the PMOS and Pain cohort was 33.88% compared to a 14.63% prevalence in the entire PMOS cohort. Excess abdominal visceral fat is well-documented to increase inflammation (Després, 2012), and PMOS is considered a pro-inflammatory condition linked with cardiovascular disease (CVD) and T2D. This inflammation, in turn, can underlie obesity, CVD, and insulin resistance (IR) (Abraham Gnanadass et al., 2021; Osborn and Olefsky, 2012). Our data show that 12.43% of patients with PMOS and Pain also had a diagnosis for essential hypertension, and 7.84% of PMOS and Pain patients had a T2D diagnosis. These results underscore the health challenges faced by individuals dealing with both PMOS and Pain issues, necessitating treatment approaches that address both the syndrome itself and its accompanying symptoms.

Women with PMOS are significantly at risk for future health outcomes such as infertility, T2D, coronary heart disease, dyslipidemia, depression, non-alcoholic fatty liver disease, and obstructive sleep apnea (Anagnostis et al., 2018; Ávila et al., 2014; Chaudhuri, 2023; McGowan, 2011; Patel, 2018; Zore et al., 2017). Our results also highlight specific risks for different subgroups (PMOS overall and PMOS and Pain). In the overall PMOS cohort, the highest future health outcome risks are for infertility (RR = 3.54) and T2D (RR = 2.77). However, in patients with PMOS and Pain, the highest risks are for ovarian cysts (RR = 2.23). Ovarian cysts are a hallmark feature of polycystic ovarian morphology (PCOM), which is caused by immature/arrested follicles that do not ovulate and cause a ‘string of pearls’ appearance and enlarging of the ovaries (Adashi et al., 2023; Tsilchorozidou et al., 2004). Ovarian cysts have long been disputed by the PMOS research community as not being associated with PMOS and therefore, not being associated with pain. However, the magnitude of this risk, as shown in our results, underscores the importance of regular monitoring and appropriate management strategies for patients presenting with both PMOS and pain symptoms. Liver disease had high and comparable RR in both cohorts with overall PMOS (RR = 2.23) and PMOS and Pain (RR = 1.89). PMOS is known to be linked with non-alcoholic fatty liver disease (NAFLD; Butt and Devi, 2024; Kumarendran et al., 2018; Torres and Harrison, 2016). This association between PMOS and pain and liver disease may be explained by the shared metabolic disturbances common to both PMOS and NAFLD, such as insulin resistance and dyslipidemia (Georgescu, 2022; Qu et al., 2013; Torres and Harrison, 2016). The presence of chronic pain could potentially exacerbate these metabolic imbalances through various mechanisms, including altered stress responses and lifestyle factors (Kivimäki et al., 2023). These findings suggest that patients with PMOS who also experience chronic pain may represent a distinct phenotype with unique risk profiles. In contrast, women with PMOS without documented pain demonstrated higher relative risks for infertility, obesity, and T2D, suggesting a more metabolically driven PMOS phenotype. The differing RR patterns between PMOS with and without pain may therefore reflect heterogeneity in underlying pathophysiology, symptom recognition, or healthcare utilization. Further longitudinal and mechanistic studies will be needed to better understand these distinct clinical trajectories. Additionally, the increased risk for future health conditions in the PMOS and Pain cohort also suggests that pain may be an important marker for identifying individuals at risk of developing future health outcomes, necessitating more vigilant monitoring and proactive intervention.

Women with PMOS had a higher future risk of depression (RR = 1.23) and anxiety (RR = 1.11). These associations were substantially stronger in the PMOS and Pain cohort (depression RR = 1.43; anxiety RR = 1.37). This pattern aligns with prior evidence and supports routine mental health screening as part of PMOS care (Teede et al., 2018; Teede et al., 2023). Clinically, the higher RR estimates in the pain-enriched PMOS subgroup can be supported by the understanding that persistent pain associated with PMOS (dysmenorrhea, abdominal, and pelvic pain), which can amplify stress, sleep disruption, and functional impairment, all of which can worsen mood and anxiety and increase healthcare contacts where these diagnoses are captured (O’Brien and Bosak, 2025; Sai and Mahaparale, 2024). We also observed increased future GERD risk in the PMOS overall cohort (RR = 1.32), with a marked elevation in the PMOS and Pain cohort (RR = 1.80). The increased RR for GERD in women with PMOS and PMOS and Pain supports the established model that obesity and central adiposity in PMOS, particularly in those with pain, is a factor for GERD and its complications (Hampel et al., 2005). Finally, acute pharyngitis showed increased risk specifically in the PMOS and Pain cohort (RR = 1.62). This may reflect reflux-related upper airway irritation (laryngopharyngeal reflux), which has been linked to chronic pharyngitis-type presentations, as well as utilization/coding effects in a subgroup of PMOS patients with more frequent clinical encounters (Cui et al., 2024).

Lastly, our analysis explored the impact of common PMOS medications on pain management. We found that prescriptions for COCPs, metformin, and spironolactone are associated with a reduction in reported pain symptoms. Specifically, individuals who received these medications showed a 5.00% average decreased prevalence of pain diagnoses after treatment, suggesting that these medications may also manage pain symptoms in individuals with PMOS. A recent publication looked at the association of PMOS-related medications with adverse drug reactions (ADRs) for women with PMOS and found that metformin and COCPs were significantly associated with abdominal pain (Sidra et al., 2019). However, this study did not measure the association of ADRs with pain before and after the prescription of PMOS medication. Our results offer insights for application showing that efficient pharmacological management of PMOS symptoms can also help alleviate associated pain. Additionally, the efficacy of these medications in reducing pain, specifically spironolactone and COCPs, which are prescribed in PMOS for their antiandrogenic effects, may suggest hyperandrogenism to be a contributor to increased pain in PMOS, and a potential target for addressing pain in PMOS. Furthermore, the advantages of these treatments may be beneficial, not just in managing typical PMOS symptoms, but also in tackling the significant burden of pain experienced by many women with PMOS, highlighting a valuable role in drug repurposing.

Our study has limitations that need to be considered when interpreting the results. Firstly, relying on ICD codes to identify pain and other health outcomes may not capture the range of experiences and clinical intricacies. While these codes offer an approach to data collection, they might not fully reflect variations in pain severity or the personal experiences of those with PMOS (Kataria and Ravindran, 2020). Although extensive, the use of EHR data may still contain gaps or discrepancies that could impact the accuracy of our results (Madden et al., 2016). Furthermore, since this study is observational, by nature it cannot establish a causal relationship between PMOS, pain, and future health outcomes. Moreover, the demographic variations observed—especially the higher occurrence of pain among individuals—could be influenced by socio-economic factors, access to healthcare, nutrition, and other unmeasured variables. In addition, self-reported race was not captured the same globally as it is not a variable that is coded by HCOs worldwide. Lastly, TriNetX captures only medication prescriptions, which does not allow our analysis to consider adherence issues, dosage differences, or concurrent treatments that may influence the outcomes observed.

We were unable to evaluate the use of analgesics or anti-inflammatory medications, as over-the-counter pain medications commonly used for dysmenorrhea and pelvic pain are not consistently captured within the TriNetX electronic health record system. This limitation prevents the assessment of how pain-specific treatments may influence reported pain outcomes.

Future research should focus on overcoming these limitations through studies with detailed clinical assessments and a broader range of demographic and socio-economic factors.

### Conclusion

Various pain subtypes can profoundly affect the daily lives of PMOS patients. Due to limited research in clinical and laboratory settings, the effects and underlying mechanisms of pain remain unclear. Our study highlights the significant prevalence and impact of pain in women with PMOS, revealing critical differences across racial groups and underscoring the heightened risk for future health complications in those experiencing pain. These findings emphasize the importance of comprehensive pain assessment, management, and inclusion as guidelines in the standard care of PMOS, with a particular focus on addressing racial disparities. Additionally, the observed effectiveness of medications such as systemic oral contraceptives, metformin, and spironolactone in reducing pain symptoms provides valuable insights for clinical practice, suggesting that these treatments can offer dual benefits in managing both PMOS and associated pain. Dysmenorrhea, abdominal, and pelvic pain are common experiences in women with PMOS, in the absence of pelvic-related conditions that can contribute to this type of pain, such as pelvic inflammatory disease, endometriosis, and fibroids. It is crucial to distinguish between pain originating from PMOS and Pain arising from comorbidities to ensure appropriate management and targeted treatment strategies for improving the quality of life in affected individuals.

### Compliance with ethical standards

The data available on the TriNetX platform is de-identified and no personal health information (PHI) is shared with users. Data accessed through the TriNetX platform is therefore not considered ‘human subjects research’ according to Section §164.514(b)(1) of the HIPAA Privacy Rule and is exempt from IRB approval.

## Data Availability

All summary-level data has been included in this manuscript in Tables 2–4, Figure 3—source data 1 and 2. For reproducibility, detailed methods are described in the Methods section of this manuscript including all diagnosis and covariate codes. Raw data cannot be made publicly available as the de-identified individual-level human subject data is accessible on the TriNetX Global Network workspace. Researchers (both academic and commercial) interested in working with the TriNetX Global Network data can visit the TriNetX website (https://trinetx.com/) to learn more about getting access or contact support@trinetx.com to receive direct support from the TriNetX team. No IRB approval is needed as the data is de-identified and no personal health information is shared with users.

## Additional files

MDAR checklist
