# Supplementary material for: Understanding pain in women with polyendocrine metabolic ovarian syndrome: health risks and treatment effectiveness
Source: eLife. 2026 Aug 28;14:RP103875. doi: 10.7554/eLife.103875 (PMC13524376; doi:10.7554/eLife.103875)
Supplement: Supplementary file 1 [file elife-103875-fig3-data1.docx]

| **Figure 3-source data 1.** Relative risk ratios (RR) for race-stratified PMOS and Pain cohorts. | | | | |
| --- | --- | --- | --- | --- |
| Outcomes | RR | 95 % CI Lower | 95 % CI Upper | RACE |
| Acute Pharyngitis | 1.48116696 | 1.29900096 | 1.68887908 | Asian |
| Acute Pharyngitis | 1.66642937 | 1.57489338 | 1.76328562 | B/AA |
| Acute Pharyngitis | 1.60569876 | 1.48306611 | 1.73847174 | Other |
| Acute Pharyngitis | 1.53787092 | 1.49711267 | 1.57973878 | White |
| Anxiety | 1.37047289 | 1.21289211 | 1.5485268 | Asian |
| Anxiety | 1.44386081 | 1.38026336 | 1.5103886 | B/AA |
| Anxiety | 1.42629925 | 1.3311973 | 1.52819537 | Other |
| Anxiety | 1.36169361 | 1.33334855 | 1.39064125 | White |
| Depression | 1.47187919 | 1.26181799 | 1.71691033 | Asian |
| Depression | 1.53080853 | 1.45255841 | 1.61327403 | B/AA |
| Depression | 1.58435711 | 1.4575857 | 1.72215428 | Other |
| Depression | 1.42379325 | 1.38739798 | 1.46114328 | White |
| Essential Hypertension | 1.10175221 | 0.92689833 | 1.30959124 | Asian |
| Essential Hypertension | 1.18313939 | 1.11944727 | 1.25045534 | B/AA |
| Essential Hypertension | 1.06165101 | 0.96025234 | 1.17375696 | Other |
| Essential Hypertension | 1.18741942 | 1.1492195 | 1.22688909 | White |
| GERD | 1.86396399 | 1.60692434 | 2.16211906 | Asian |
| GERD | 1.68644514 | 1.59424661 | 1.78397571 | B/AA |
| GERD | 1.9067563 | 1.74527112 | 2.08318328 | Other |
| GERD | 1.73962561 | 1.69145936 | 1.78916345 | White |
| Infertility | 0.92789088 | 0.77790505 | 1.10679508 | Asian |
| Infertility | 1.22993058 | 1.12907207 | 1.33979864 | B/AA |
| Infertility | 1.34484386 | 1.17822233 | 1.53502863 | Other |
| Infertility | 0.96529958 | 0.92097876 | 1.01175329 | White |
| Kidney Disease | 0.80568333 | 0.51015392 | 1.27241132 | Asian |
| Kidney Disease | 1.25078401 | 1.05720935 | 1.47980213 | B/AA |
| Kidney Disease | 1.20232146 | 0.85431738 | 1.69208415 | Other |
| Kidney Disease | 1.55051184 | 1.3989211 | 1.71852934 | White |
| Liver Disease | 1.7390311 | 1.44298843 | 2.09580973 | Asian |
| Liver Disease | 2.06281014 | 1.84566266 | 2.30550564 | B/AA |
| Liver Disease | 1.72535256 | 1.52806526 | 1.94811147 | Other |
| Liver Disease | 1.85267848 | 1.77495717 | 1.93380302 | White |
| Obesity | 1.19010005 | 1.06383317 | 1.33135362 | Asian |
| Obesity | 1.04902361 | 1.00414517 | 1.09590781 | B/AA |
| Obesity | 1.10151702 | 1.02825194 | 1.1800024 | Other |
| Obesity | 1.11370946 | 1.08908507 | 1.13889061 | White |
| Ovarian Cysts | 1.63305195 | 1.34853662 | 1.97759455 | Asian |
| Ovarian Cysts | 2.27846823 | 2.10514611 | 2.46606041 | B/AA |
| Ovarian Cysts | 2.31263825 | 2.05642382 | 2.600775 | Other |
| Ovarian Cysts | 2.14690293 | 2.06404786 | 2.23308397 | White |
| T2D | 0.97408555 | 0.82056074 | 1.15633444 | Asian |
| T2D | 1.11663425 | 1.04370298 | 1.19466177 | B/AA |
| T2D | 1.08770207 | 0.97431501 | 1.21428468 | Other |
| T2D | 1.12329217 | 1.07820896 | 1.17026043 | White |
