# Supplementary material for: Understanding pain in women with polyendocrine metabolic ovarian syndrome: health risks and treatment effectiveness
Source: eLife. 2026 Aug 28;14:RP103875. doi: 10.7554/eLife.103875 (PMC13524376; doi:10.7554/eLife.103875)
Supplement: Supplementary file 2 [file elife-103875-fig3-data2.docx]

| **Figure 3-source data 2.** Significance for relative risk ratios for each self-reported race combination. Both P-value (P) and adjusted p-values using Benjamini-Hochberg (P(BH)) are reported. Significant differences in RR between self-reported race groups are bolded. | | | | | |
| --- | --- | --- | --- | --- | --- |
| Future Health Outcome | Group 1 | Group 2 | Z | P | P(BH) |
| Acute Pharyngitis | Asian | B/AA | -1.6166987 | 0.10594334 | 0.31783001 |
| Acute Pharyngitis | Asian | Other | -1.0314123 | 0.3023475 | 0.46968711 |
| Acute Pharyngitis | Asian | White | -0.5496948 | 0.58252871 | 0.58252871 |
| Acute Pharyngitis | B/AA | Other | 0.74639393 | 0.45542949 | 0.54651538 |
| Acute Pharyngitis | B/AA | White | 2.5154627 | 0.01188763 | 0.07132575 |
| Acute Pharyngitis | Other | White | 1.00868706 | 0.31312474 | 0.46968711 |
| Anxiety | Asian | B/AA | -0.7853388 | 0.43225496 | 0.86450992 |
| Anxiety | Asian | Other | -0.5578196 | 0.57696755 | 0.86545132 |
| Anxiety | Asian | White | 0.10162617 | 0.91905341 | 0.91905341 |
| Anxiety | B/AA | Other | 0.29106415 | 0.77100226 | 0.91905341 |
| Anxiety | B/AA | White | 2.30990701 | 0.0208933 | 0.12535982 |
| Anxiety | Other | White | 1.25941664 | 0.20787988 | 0.62363965 |
| Depression | Asian | B/AA | -0.4729643 | 0.63623868 | 0.676734 |
| Depression | Asian | Other | -0.8241903 | 0.40983142 | 0.676734 |
| Depression | Asian | White | 0.41692399 | 0.676734 | 0.676734 |
| Depression | B/AA | Other | -0.6839542 | 0.49400409 | 0.676734 |
| **Depression** | **B/AA** | **White** | **2.42762719** | **0.01519796** | **0.04940984** |
| **Depression** | **Other** | **White** | **2.39833165** | **0.01646995** | **0.04940984** |
| Essential Hypertension | Asian | B/AA | -0.7698166 | 0.44140868 | 0.66211302 |
| Essential Hypertension | Asian | Other | 0.36361672 | 0.71614424 | 0.85937309 |
| Essential Hypertension | Asian | White | -0.8344677 | 0.4040175 | 0.66211302 |
| Essential Hypertension | B/AA | Other | 1.8526185 | 0.06393706 | 0.19181118 |
| Essential Hypertension | B/AA | White | -0.1101123 | 0.91232028 | 0.91232028 |
| Essential Hypertension | Other | White | -2.0784656 | 0.0376665 | 0.19181118 |
| GERD | Asian | B/AA | 1.23623073 | 0.21637281 | 0.43274562 |
| GERD | Asian | Other | -0.2575035 | 0.79679012 | 0.79679012 |
| GERD | Asian | White | 0.89599097 | 0.37025759 | 0.4443091 |
| GERD | B/AA | Other | -2.2953454 | 0.02171334 | 0.13028004 |
| GERD | B/AA | White | -0.9683209 | 0.33288412 | 0.4443091 |
| GERD | Other | White | 1.93661501 | 0.05279242 | 0.15837727 |
| **Infertility** | **Asian** | **B/AA** | **-2.8183597** | **0.00482697** | **0.00724046** |
| **Infertility** | **Asian** | **Other** | **-3.3001771** | **0.00096624** | **0.00193248** |
| Infertility | Asian | White | -0.4245574 | 0.67115934 | 0.67115934 |
| Infertility | B/AA | Other | -1.111312 | 0.26643407 | 0.31972089 |
| **Infertility** | **B/AA** | **White** | **4.86428327** | **1.15E-06** | **6.89E-06** |
| **Infertility** | **Other** | **White** | **4.62996514** | **3.66E-06** | **1.10E-05** |
| Kidney Disease | Asian | B/AA | -1.7704376 | 0.07665427 | 0.15330853 |
| Kidney Disease | Asian | Other | -1.3750696 | 0.16910986 | 0.20293183 |
| **Kidney Disease** | **Asian** | **White** | **-2.7392558** | **0.00615784** | **0.03694707** |
| Kidney Disease | B/AA | Other | 0.2033752 | 0.83884178 | 0.83884178 |
| Kidney Disease | B/AA | White | -2.1359645 | 0.03268231 | 0.09804693 |
| Kidney Disease | Other | White | -1.3968767 | 0.16245065 | 0.20293183 |
| Liver Disease | Asian | B/AA | -1.5404237 | 0.12345711 | 0.24691422 |
| Liver Disease | Asian | Other | 0.06951799 | 0.94457731 | 0.94457731 |
| Liver Disease | Asian | White | -0.6480213 | 0.51697119 | 0.62036542 |
| Liver Disease | B/AA | Other | 2.12621006 | 0.03348577 | 0.20091464 |
| Liver Disease | B/AA | White | 1.76655909 | 0.0773021 | 0.23190629 |
| Liver Disease | Other | White | -1.083748 | 0.27847656 | 0.41771484 |
| Obesity | Asian | B/AA | 2.05439618 | 0.03993736 | 0.11981209 |
| Obesity | Asian | Other | 1.15206197 | 0.24929561 | 0.30666863 |
| Obesity | Asian | White | 1.13695477 | 0.25555719 | 0.30666863 |
| Obesity | B/AA | Other | -1.1736814 | 0.24052266 | 0.30666863 |
| Obesity | B/AA | White | -2.3881842 | 0.01693185 | 0.10159113 |
| Obesity | Other | White | -0.2981351 | 0.76560009 | 0.76560009 |
| **Ovarian Cysts** | **Asian** | **B/AA** | **-3.1514691** | **0.00162451** | **0.00717584** |
| **Ovarian Cysts** | **Asian** | **Other** | **-3.0366859** | **0.00239195** | **0.00717584** |
| **Ovarian Cysts** | **Asian** | **White** | **-2.7436718** | **0.00607562** | **0.01215125** |
| Ovarian Cysts | B/AA | Other | -0.2060601 | 0.83674398 | 0.83674398 |
| Ovarian Cysts | B/AA | White | 1.31921453 | 0.1870974 | 0.2806461 |
| Ovarian Cysts | Other | White | 1.17691992 | 0.23922747 | 0.28707296 |
| T2D | Asian | B/AA | -1.4522022 | 0.1464454 | 0.43933621 |
| T2D | Asian | Other | -1.0609978 | 0.28869092 | 0.57738184 |
| T2D | Asian | White | -1.5841392 | 0.11316204 | 0.43933621 |
| T2D | B/AA | Other | 0.39837874 | 0.69035103 | 0.82842123 |
| T2D | B/AA | White | -0.147501 | 0.88273658 | 0.88273658 |
| T2D | Other | White | -0.5372402 | 0.59110168 | 0.82842123 |
