# Supplementary material for: Understanding pain in women with polyendocrine metabolic ovarian syndrome: health risks and treatment effectiveness
Source: eLife. 2026 Aug 28;14:RP103875. doi: 10.7554/eLife.103875 (PMC13524376; doi:10.7554/eLife.103875)
Supplement: Peer review history

# Editor report: eLife Assessment

**DOI:** 10.7554/eLife.103875.3.sa0

**Authors:** Daria Lizneva

**Affiliations:** Icahn School of Medicine at Mount Sinai United States

**Keywords:** Compelling, Valuable

This study presents **valuable** findings on the high prevalence of pain in women with polycystic ovary syndrome and its association with distinct future health risks across different racial groups. The evidence supporting the conclusions is **compelling**, utilizing a massive global dataset and rigorous propensity score matching to identify pain as a critical, yet underexplored, clinical marker. The work will be of interest to reproductive endocrinologists, medical biologists, and clinicians involved in the diagnosis and management of polycystic ovary syndrome.

# Referee report: Reviewer #1 (Public review)

**DOI:** 10.7554/eLife.103875.3.sa1

Summary:

This retrospective study provides a new data regarding the prevalence of pain in women with PCOS and its relationship with health outcomes. Using data from electronic health records (EHR), the authors found a significantly higher prevalence of pain among women with PCOS compared to those without the condition: 19.21% of women with PCOS versus 15.8% in non-PCOS women. The highest prevalence of pain was conducted among Black or African American (32.11%) and White (30.75%) populations. Besides, women with PCOS and pain have at least a 2-fold increased prevalence of obesity (34.68%) at baseline compared to women with PCOS in general (16.11%). Also, women with PCOS had the highest risk for infertility and T2D, but women with PCOS and pain had higher risks for ovarian cysts and liver disease. Regarding these results, authors suggested the critical need to address pain in the diagnosis and management of PCOS due to its significant impact on patient health outcomes.

Strengths:

The problem of pain assessment in PCOS patients is well described and authors provided a clear rationale selection of the retrospective design to investigate this problem.

A large number of analyzed patient's records (76,859,666 women) and its uniformity increases the power of the study. Using the Propensity Score Matching makes possible to reduce the heterogeneity of the compared cohorts and influence of comorbid conditions.

Analysis in different ethnic cohorts provides actual and necessary data regarding the prevalence of pain and its relationship with different health conditions that will be helpful for clinicians to make a diagnosis and manage the PCOS in women of different ethnicity.

Assessment of risk of different health conditions as including PCOS-associated pathology as other common groups of diseases in PCOS women with or without pain allows to differentiate the risk of comorbid conditions depending on the presence of one symptom (pelvic or abdominal pain, dysmenorrhea).

Weaknesses:

The significant weakness of the study is the absence of Latin American cohort. Probably the White cohort includes Latin Americans or others, but results of the study cannot be extrapolated to particular White ethnicities.

Comments on revised version:

At present, I have no questions or recommendations for the authors, as they have exhaustively addressed the previous comments and incorporated the necessary corrections.

# Referee report: Reviewer #2 (Public review)

**DOI:** 10.7554/eLife.103875.3.sa2

Summary:

The study offers a thorough analysis of the prevalence of pain in women with polycystic ovary syndrome (PCOS) and its associations with health outcomes across various racial groups. Furthermore, the research investigates the prevalence of PCOS and pain among different racial demographics, as well as the increased risk of developing various conditions in comparison to individuals who have PCOS alone.

Strengths:

The study emphasizes pain as a significant comorbidity of PCOS, an area that is critically underexplored in existing literature. The findings regarding the increased prevalence of some of the diseases in the PCOS + pain group provide valuable direction for future research and clinical care. I believe physicians should incorporate pain score assessments into their clinical practice to improve patients' quality of life and raise awareness about pain management. If future research focuses on the mechanisms of pain, it would provide a better understanding of pain and allow for a focus on the underlying causes rather than just symptomatic management. The study also highlights the association between PCOS+pain and various comorbidities, such as obesity, hypertension, and type 2 diabetes, as well as conditions like infertility and ovarian cysts, offering a holistic view of the burden of PCOS.

Weaknesses:

Due to the nature of retrospective design, some data may not be readily available in the EHR system. Diagnosis of PCOS, pain is based on ICD codes, which may lead to misclassification and may not capture symptom severity or patient-reported experiences.

# Author comment: Author response

**DOI:** 10.7554/eLife.103875.3.sa3

**Authors:** Tess Cherlin, Stephanie Mohammed, Samantha Strydesky, Sasha Ottey, Katherine Sherif, Shefali Setia Verma

**Affiliations:** University of Pennsylvania PA United States; University of Pennsylvania PA United States; University of Pennsylvania Philadelphia United States; PCOS Challenge Inc Georgia United States; Thomas Jefferson University PA United States; University of Pennsylvania PA United States

The following is the authors’ response to the original reviews.

**Public Reviews:**

**Reviewer #1 (Public review):**

Summary:

This retrospective study provides new data regarding the prevalence of pain in women with PCOS and its relationship with health outcomes. Using data from electronic health records (EHR), the authors found a significantly higher prevalence of pain among women with PCOS compared to those without the condition: 19.21% of women with PCOS versus 15.8% in non-PCOS women. The highest prevalence of pain was conducted among Black or African American (32.11%) and White (30.75%) populations. Besides, women with PCOS and pain have at least a 2-fold increased prevalence of obesity (34.68%) at baseline compared to women with PCOS in general (16.11%). Also, women with PCOS had the highest risk for infertility and T2D, but women with PCOS and pain had higher risks for ovarian cysts and liver disease. Regarding these results, the authors suggested the critical need to address pain in the diagnosis and management of PCOS due to its significant impact on patient health outcomes.

Strengths:

(1) The problem of pain assessment in PCOS patients is well described and the authors provided a clear rationale selection of the retrospective design to investigate this problem.

(2) A large number of analyzed patient records (76,859,666 women) and their uniformity increases the power of the study. Using the Propensity Score Matching makes it possible to reduce the heterogeneity of the compared cohorts and the influence of comorbid conditions.

(3) Analysis in different ethnic cohorts provides actual and necessary data regarding the prevalence of pain and its relationship with different health conditions that will be helpful for clinicians to make a diagnosis and manage PCOS in women of different ethnicities.

(4) Assessment of the risk of different health conditions including PCOS-associated pathology as other common groups of diseases in PCOS women with or without pain allows to differentiate the risk of comorbid conditions depending on the presence of one symptom (pelvic or abdominal pain, dysmenorrhea).

We would like to thank the Reviewer for their positive feedback on this manuscript. Pain assessment in women with PCOS is of paramount interest and because of a gap in this research area, we are trying to address it.

Weaknesses:

(1) Although the paper has strengths in methodology and data analysis, it also has some weaknesses. The lack of a hypothesis doesn't allow us to evaluate the aim and significance of this study.

We would like to thank the Reviewer for their valuable feedback regarding the hypothesis of this study. We understand that the hypothesis may not have been written clearly under the objectives and we have corrected this in the formal revision.

The primary hypothesis of this study is that women with PCOS experience a higher prevalence to pain (including dysmenorrhea, abdominal pain and pelvic pain) compared to women without PCOS, and this prevalence varies by racial groups. Our hypothesis aims to explore the relationship between PCOS and pain, the associated health risks, and the potential racial disparities in pain prevalence and long-term health outcomes. Additionally, we seek to assess the effect of treatment on reducing pain symptoms in women with PCOS. This study not only examines the immediate burden of pain but also investigates its long-term consequences, including risks of infertility, obesity, and type 2 diabetes.

To enhance clarity for readers, we explicitly stated this hypothesis in the revised manuscript and have ensured that its connection to the study’s objectives is clearly articulated. We appreciate the Reviewer’s insights and have incorporated these refinements to strengthen the manuscript.

(2) The exclusion criteria don't include conditions, that can lead to symptoms similar to PCOS: thyroid diseases, hyperprolactinemia, and congenital adrenal hyperplasia. Thyroid status is not being taken into account in the criteria for matching. All these conditions could occur as on prevalence results as on risk assessment.

We would like to thank the Reviewer for highlighting the need to include these additional conditions that mimic PCOS. After excluding hypothyroidism, hyperprolactinemia, and adrenal hyperplasia from the PCOS and PCOS and pain cohorts, we observed that 7,690 patients (1.65%) with PCOS and 1,854 patients (1.36%) with PCOS were removed. Based on this observation, we added these three conditions to our exclusion criteria and reran all our analysis for disease for our resubmission. The manuscript, figures, and tables have been updated to reflect these exclusions. Additionally, we have added rationale for excluding these conditions to the Discussion. With these major changes to the analysis, we aim to improve transparency and provide more accurate results and precise interpretations of our findings to the field.

(3) The significant weakness of the study is the absence of a Latin American cohort. Probably the White cohort includes Latin Americans or others, but the results of the study cannot be extrapolated to particular White ethnicities.

We appreciate the Reviewer’s suggestion to include Latin American cohorts in this study. The TriNetX platform has both self-reported race and ethnicity demographic information. In Table 3 - Figure Supplement 5 and Table 4 - Figure Supplement 6 we include baseline demographic information for both race (Asian, Black or African American, Native Hawaiian or Other Pacific Islander, Other, White, and Unknown Race) and ethnicity (Not Hispanic or Latino, Unknown, and Hispanic or Latino). In this paper we focused our future health outcome sub-analysis on four self-reported race groups: Asian, Black or African American, Other (Native Hawaiian or Other Pacific Islander, Other, Unknown Race), and White. We agree that including Latin American cohorts in the analysis is essential to better understand the health disparities affecting this population. Future work to better define Latin American cohorts in EHR data would significantly aid our ability to investigate this further.

(4) The authors didn't provide sufficient rationale for future health outcomes and this list didn't include diseases of the digestive system or disorders of thyroid glands, which can also cause abdominal pain.

We appreciate the Reviewer comment and concern regarding additional rationale for future health outcomes. We originally chose to investigate general future health outcomes like disease of the digestive system, circulatory system, etc. These disease groups were selected based on being general and having high prevalence as future health outcomes for patients with PCOS and Pain.

Our initial results highlight the prevalence of disorders of the digestive system (Figure 2). However, after considering the Reviewers comments and to further strengthen our analysis, we included the most prevalent digestive system disorder in our relative risk (RR) analysis. Gastro-esophageal reflux disease (GERD) was identified as the most prevalent future digestive condition for women with PCOS and Pain (13.5%). There was also a 10.5% prevalence in women with PCOS overall.

We were not able to include the same analysis for thyroid dysfunctions as this condition is a part of our exclusion criterion. These updates have been incorporated into the revised manuscript to ensure clarity and completeness.

**Reviewer #2 (Public review):**

Summary:

The study offers a thorough analysis of the prevalence of pain in women with polycystic ovary syndrome (PCOS) and its associations with health outcomes across various racial groups. Furthermore, the research investigates the prevalence of PCOS and pain among different racial demographics, as well as the increased risk of developing various conditions in comparison to individuals who have PCOS alone.

Strengths:

The study emphasizes pain as a significant comorbidity of PCOS, an area that is critically underexplored in existing literature. The findings regarding the increased prevalence of some of the diseases in the PCOS + pain group provide valuable direction for future research and clinical care. I believe physicians should incorporate pain score assessments into their clinical practice to improve patient's quality of life and raise awareness about pain management. If future research focuses on the mechanisms of pain, it would provide a better understanding of pain and allow for a focus on the underlying causes rather than just symptomatic management. The study also highlights the association between PCOS+pain and various comorbidities, such as obesity, hypertension, and type 2 diabetes, as well as conditions like infertility and ovarian cysts, offering a holistic view of the burden of PCOS.

We sincerely appreciate the Reviewer’s insightful comments. We hope that our findings will encourage further research on the occurrence of pain in women with PCOS and that others will replicate our results to strengthen the evidence in this area. As noted in our introduction, there are currently no standardized abdominal pain score assessments specifically for women with PCOS. We hope that the findings from this study will contribute to efforts toward developing a standardized pain assessment for the PCOS community. In the meantime, further research across more diverse populations will be essential to build a more comprehensive understanding of this issue.

Weaknesses:

Due to the nature of the retrospective study, some data may not be readily available in the system. Instead of simply categorizing participants based on whether they experience pain, it would be more useful to employ a pain scale or questionnaire to better understand the severity and type of patients' pain. This approach would allow for a more thorough analysis of pain improvement following treatment with the three widely used medications for PCOS. Additionally, it would be beneficial for the authors to specify subtypes of the disease rather than generalizing conditions, such as mentioning specific digestive system disorders or mental health disorders. The lack of detailed analysis of specific disorders limits the depth of the findings. This may cause authors to make incorrect conclusions.

We appreciate the Reviewer for highlighting the importance of categorizing pain levels experienced by women with PCOS. However, there is currently no standardized pain assessment for abdominal pain, and therefore more research is required before such a classification can be made. Additionally, the electronic health record data we leveraged via the TriNextX platform does not include any pain scale data from unstructured notes. Despite these limitations, this study is an important step toward recognizing abdominal and pelvic pain in women with PCOS. Our findings indicate that women with PCOS report abdominal pain independent of digestive conditions such as irritable bowel syndrome— a condition often associated with pain in this population.

We would like to thank the Reviewer for their thoughtful comment with respect to subtyping future health outcomes. To get at the most impactful future health outcomes affecting women with PCOS and Pain, we have included the top 5 most prevalent health outcomes associated with PCOS and Pain. Specifically, we included analysis for anxiety disorder, depressive episodes, essential hypertension, Gastro-esophageal reflux disease (GERD), and acute pharyngitis. We observed that 17.1%, 11.5%, 10.5%, 10.0% of patients with PCOS and 20.1%, 13.7%, 13.5%, 13.3% of patients with PCOS and Pain were at risk of developing anxiety, depression, acute pharyngitis, and GERD respectively. For our revision, we have included these 5 conditions in our PCOS, PCOS and Pain and self-reported race-stratified future health outcome relative risk (RR) analyses. The revised manuscript, figures, and tables all reflect these changes.

**Recommendations for the authors:**

**Reviewer #1 (Recommendations for the authors):**

(1) I highly recommend checking all papers and supplements for misprints. There are a lot of missing spaces in the Introduction.

We would like to thank the Reviewer for bringing this to our attention. We have carefully reviewed the manuscript and all supplementary materials and corrected formatting issues, including missing spaces and typographical errors throughout the Introduction and the rest of the document.

(2) Supplementary Table 3: numbers from the first line in "%No PCOS" should be in "No PCOS"?

We thank the Reviewer for bringing this error to our attention. We have identified the source of the problem and values have been added to the appropriate column.

(3) Why for the matching authors use the categorical data for overweight/obesity and not the entire values? There are different stages of obesity that can be predominant in different cohorts and contribute to the results.

We would like to thank the Reviewer for their insightful question. While TriNetX does have some BMI values for patient participants, this data is not included for all patients. For example, only 29-30% of women in the PCOS control and case cohorts have BMI recorded. Therefore, we focused on ICD codes for obesity instead to include as much data as possible.

(4) What criteria were being used to determine hyperlipidemia and obesity? Were these criteria equal for all patients, or did they depend on ethnicity?

We would like to apologize to the Reviewer for any confusion. The criteria to determine hyperlipidemia and obesity are ICD-10-CM codes as recorded in the TriNetX platform. The ICD-10-CM codes for obesity are E65-E68 and the ICD-10-CM code for hyperlipidemia is E78.5. Please also see the Methods section of this manuscript where all the ICD-10-CM codes are described.

(5) The section material and methods should provide information regarding quality assurance checks and any steps to eliminate data suspected to be unreliable or invalid, to process missing data, consisting of data or claim duplicates. If quality assurance of data hadn't been conducted, it should have been noticed in the study limitations.

We thank the Reviewer for this suggestion. We have revised the Methods section to explicitly describe the data quality assurance procedures inherent to the TriNetX platform. Specifically, we clarified that TriNetX applies standardized data mapping to controlled clinical terminologies (ICD, CPT, RxNorm), performs automated quality checks and excludes records that do not meet platform-defined standards.

(6) It's not clear why the authors didn't include in the analysis the information regarding taking painkillers or anti-inflammatory drugs by patients. Maybe there is no such data in EHR. However, if the patient has some chronic inflammatory or autoimmune disease, she should be prescribed medication. I recommend specifying this issue in the section Material and Methods and/or study limitations.

We would like to thank the Reviewer for this important suggestion. We have now clarified this point in the limitations section of the discussion. Specifically, we added text explaining that over-the-counter analgesics and anti-inflammatory medications are not reliably captured by EHR or within the TriNetX platform and therefore could not be evaluated in our analysis.

(7) The authors should provide the Table or complete Supplementary Tables 2 and 3 with the parameters of patients used for matching.

We apologize to the Reviewer for any confusion. The parameters used for propensity score matching are described fully in the Methods section of the paper. Table 2 – Figure Supplement 5 and Table 3 – Figure supplement 6 display baseline characteristics for patients before and after the 1:1 propensity score matching using these parameters. We have now also added the propensity score matching parameters to the table descriptions to provide fluidity and further clarification.

(8) The authors found out that women with PCOS and pain have higher RR for ovary cysts and liver diseases compared to women with PCOS who have higher RR for infertility, obesity, and T2D. Discussion includes thoughts regarding a higher risk of ovary cysts and liver disease in women with PCOS and pain, but there is not any suggestion as to why women with PCOS and without pain have a higher risk of infertility, obesity, and T2D. If there is no data explaining this phenomenon, I recommend noting the need for additional research.

We would like to thank the Revier for this helpful feedback. The Discussion section now includes deeper insights into the pathophysiology behind the two distinct PCOS phenotypes (PCOS overall vs. PCOS and Pain) and their differing risk profiles for future health outcomes. Specifically, we note that while women with PCOS overall may be more metabolically driven (higher risk of infertility, obesity, and T2D), women with PCOS and Pain show a higher risk of ovarian cysts and liver disease. We clarify that these findings are observational and hypothesis-generating and emphasize the need for future longitudinal and mechanistic studies.

(9) The authors suggested that systematic contraceptives, metformin, or spironolactone reduce pain in PCOS women. The reduction is significant, but the number of patients with beneficial effects is low (2.5-7.5%). Is it enough to recommend prescribing this medication not only for PCOS treatment but against pain?

We thank the Reviewer for this important comment. We agree that although the reduction in pain diagnoses following treatment with COCPs, metformin, or spironolactone was statistically significant, the absolute proportion of patients experiencing benefit was modest. Our intention was not to recommend prescribing these medications solely for pain management, but rather to highlight that standard PCOS therapies may have additional benefits in reducing pain symptoms. We have clarified this point in the Discussion to emphasize that these findings are observational and hypothesis-generating, and that prospective studies are needed before these medications can be considered specifically for pain management in PCOS.

**Reviewer #2 (Recommendations for the authors):**

(1) Including a subtype analysis of specific diseases on digestive, respiratory, and mental health diseases rather than generalizing the system will enhance the content.

We would like to thank the Reviewer for this helpful suggestion. In the revised manuscript, instead of the generalized disease systems we previously reported on, we have included analysis for the top 5 most prevalent conditions. Specifically, we included analysis for anxiety disorder, depressive episodes, essential hypertension, Gastro-esophageal reflux disease (GERD), and acute pharyngitis. We observed that 17.1%, 11.5%, 10.5%, 10.0% of patients with PCOS and 20.1%, 13.7%, 13.5%, 13.3% of patients with PCOS and Pain were at risk of developing anxiety, depression, acute pharyngitis, and GERD respectively.

(2) Including the prevalence of dysmenorrhea among healthy populations would allow readers to better compare its impact on the lives of individuals with PCOS.

We would like to apologize to the Reviewer for any confusion. The prevalence of dysmenorrhea for cases and control cohorts can be found in Table 2 – Figure Supplement 5 and Table 3 – Figure Supplement 6 before and after propensity score matching.

(3) Introducing an analysis of age subgroups will provide readers with a clearer understanding of the prevalence of pain and specific diseases across different age groups.

We would like to thank the Reviewer for this helpful suggestion. For this revision, we did a sub-analysis to explore the prevalence of PCOS and PCOS and Pain stratified by 10-year age groups. A barplot of these results can be found in Figure 4 - Figure Supplement 7.

Thank you again to the Reviewers for the positive and constructive feedback for this manuscript. We have made the appropriate edits and changes to the final revisions of the manuscript.
